# Anti-*Candida albicans* effect of the new antifungal compound Venetin-1 obtained from earthworms versus amphotericin B

**DOI:** 10.1038/s41598-026-52961-2

**Published:** 2026-05-30

**Authors:** Kinga Lewtak, Magdalena Dryglewska, Sylwia Mieszawska, Ewa Skwarek, Paulina Czaplewska, Weronika Ścibek-Rejmontowska, Hüseyin Korkmaz, Jerzy Wydrych, Wojciech Kaźmierski, Krzysztof Skrzypiec, Marta J. Fiołka

**Affiliations:** 1 Department of Immunobiology, Institute of Biological Sciences, Maria Curie-Skłodowska University, Lublin, Poland; 2 Department of Rheumatology and Connective Tissue Diseases, Medical University of Lublin, Lublin, Poland; 3 Chair and Department of Medical Microbiology, Collegium Universum, Medical University of Lublin, Lublin, Poland; 4 Department of Radiochemistry and Environmental Chemistry, Institute of Chemical Sciences, Maria Curie-Skłodowska University, Lublin, Poland; 5 Intercollegiate Faculty of Biotechnology, University of Gdańsk and Medical University of Gdańsk, Gdańsk, Poland; 6 Department of Functional Anatomy and Cytobiology, Institute of Biological Sciences, Maria Curie-Skłodowska University, Lublin, Poland; 7 Bruker Poland Sp. z o.o, Poznań, Poland; 8 Analytical Laboratory, Institute of Chemical Sciences, Maria Curie-Skłodowska University, Lublin, Poland

**Keywords:** *Candida albicans*, Venetin-1, Amphotericin B, Microscopic analysis, Flow cytometry, Proteomic analysis, Biochemistry, Biological techniques, Biotechnology, Microbiology

## Abstract

*Candida albicans* is a commensal organism that can cause disease in individuals with weakened and compromised immune systems. These fungi are responsible for superficial or invasive infections associated with high mortality. The aim of this study was to compare the activity of the new antifungal compound Venetin-1, obtained from the coelomic fluid of the earthworm *Dendrobaena veneta*, with the effects of the antibiotic amphotericin B (AmB). Flow cytometry, fluorescence microscopy, scanning electron microscopy, atomic force microscopy, and FTIR spectroscopic methods were used for comparative analysis. Venetin-1 at the highest concentration used (100 µg mL^− 1^) caused a decrease in the OD of *C. albicans* cultures by more than 60% compared to the control group, and AmB (0.5 µg mL^− 1^) by 80%. Venetin-1 at the highest concentration reduced the viability of fungal cells by 42%, while AmB reduced it by 30%. The present study shows that both Venetin-1 and AmB induced fungal cell necrosis and apoptosis. SEM analysis showed that both compounds caused morphological changes in cells, including the presence of multiple bundles at the cell poles and the formation of unseparated cell chains. Congo red fluorochrome analysis showed that both compounds caused an increase in the fluorescence intensity of the β-glucan layer in the wall. Flow cytometric analysis of the cell wall showed that Venetin-1 caused exposure of the β-glucan and chitin layers in the fungal cells. FTIR analysis confirmed molecular remodelling of the cell wall structure after exposure to both compounds. AFM analysis indicated an increase in the elasticity of the fungal cell wall after treatment with both compounds. Proteomic analysis showed that amphotericin B and Venetin-1 induced distinct proteomic response patterns in *C. albicans* cells. AmB caused concentration-restricted response detectable at sublethal conditions, whereas Venetin-1 induced a concentration-dependent and progressively expanding proteomic remodelling. The comparison of Venetin-1 and AmB revealed pronounced differences in their electrokinetic behaviour, originating from their distinct chemical structures and modes of interaction with electrolyte ions. Venetin-1 exhibited significantly more negative zeta potential values, particularly in alkaline media and in the presence of Cl⁻ ions, indicating stronger electrostatic stabilisation and higher electrokinetic stability than AmB. Considering its effect on *Candida* cells and the fact that the Venetin-1 complex has no toxic effect, this compound is a promising candidate for further biomedical research on new antifungal preparations.

**Supplementary Information:**

The online version contains supplementary material available at 10.1038/s41598-026-52961-2.

## Introduction

 Many ancient cultures, especially in Asia, used earthworms for medicinal purposes. Earthworms have been shown to contain numerous bioactive compounds that can be used as medicines. These compounds, present in coelomic fluid or extracts, have a wide range of properties, including antithrombotic and fibrinolytic^1,2^, anticancer^3–7^, antibacterial^8–11^, and antifungal^12–15^ effects. They are currently the subject of intensive biomedical research and have the potential to be used in the treatment of many conditions^16^.

For many years, our research has focused on the structure and mechanism of action of the polysaccharide-protein complex Venetin-1, obtained from the coelomic fluid (CF) of the earthworm *Dendrobaena veneta*. To date, we have demonstrated numerous properties of this compound, including antifungal activity against clinical strains of *Candida albicans*^12,13,17^, without endotoxicity or cytotoxicity to normal human skin cells, anticancer activity against A549 non-small cell lung cancer cells^5,18^, colon cancer cells^7^, and the HeLa cell line^18^ inhibition of the human 20 S proteasome^7^, and antiplatelet effects^19^.

Venetin-1 is the result of multiple processes. First, earthworms are cultured in the laboratory on a repetitive diet and maintained under the same humidity and temperature conditions. The preparation is obtained through several procedures, including isolation of CF from the earthworm body using electrostimulation, separation of coelomocytes from the fluid, microbiological filtration, heat treatment to eliminate cytotoxicity, and lyophilisation for safe sample storage. Elimination of the cytotoxicity of the coelomic fluid so that the isolated compound acts selectively on cancer and fungal cells is crucial for our research^20,21^. An important milestone in the research was the discovery that in the previously analysed fraction contained only one type of nanoparticles, which we called Venetin-1^5^ and found to be composed of fragments of lysozyme and lysenin as well as sugars^12,22^.

Amphotericin B (AmB) is a potent, broad-spectrum antifungal antibiotic often used to treat severe, life-threatening systemic fungal infections. It remains one of the most important medications in the treatment of infections caused by *Candida* species^23^. According to current data for 2026, its use is focused on invasive cases and those resistant to other antifungal drugs^24^. This drug is not recommended for use in patients with normal neutrophil counts for the treatment of non-invasive fungal infections, such as vaginal candidiasis, oral thrush, and oesophageal candidiasis. It works by damage of the fungal cell membrane but has also been associated with serious side effects. The considerable toxicity of classic amphotericin B (AmB) is associated with renal damage and electrolyte disturbances (hypokalaemia). Additionally, acute drug-related reactions, such as fever, chills, nausea, and vomiting, may occur. Research on AmB has advanced thanks to the introduction of new lipid formulations, which have a reduced nephrotoxicity profile compared to standard used formulations^25–27^. Amphotericin B is available in several forms that differ in their safety profile and use: conventional, which exhibits greater toxicity, liposomal, which is often preferred due to its lower risk of renal harm and potentially better reach of certain tissues, and the lipid complex form used in the treatment process^25–27^.

Resistance to existing drug classes - azoles and echinocandins - is rapidly increasing in species such as *Candida auris* and *Aspergillus fumigatus*, making it worthwhile to test new compounds^28^. A challenge in developing new antifungal antibiotics is that fungi have the ability to form complex biofilms on tissues and medical implants, which are nearly impenetrable to standard drug doses^29–32^. The barrier is the fact that many potential antifungal substances are poorly soluble in water^33^, which hinders the development of effective intravenous or oral formulations with good bioavailability. An additional challenge is that invasive fungal infections primarily affect critically ill or severely immunosuppressed patients, complicating trial recruitment and objective assessment of the efficacy of a new drug^34^.

The action of Venetin-1 on *C. albicans* has already been described in our previous publications; however, we have not previously compared its effects with the action of AmB, i.e. an antibiotic used as the gold standard. So far, the effect of Venetin-1 on *C. albicans* cells has been compared only to fluconazole^17^. Therefore, it seemed appropriate to conduct such an analysis, given that Venetin-1 does not demonstrate toxicity to human fibroblast cells^20^, human blood cells^19^, BEAS-2B bronchial epithelial cells^5^, or human colon epithelium CCD 841 cells^7^.

We were particularly interested in the effects of both compounds on the clinical strain of *C. albicans*. Investigating the fungal cell death pathway and structural changes seemed interesting for comparison. Such analyses are essential to determining the potential of a new antifungal compound.

## Materials and methods

###  Breeding of earthworms and isolation of Venetin-1

**Fig. 1:**
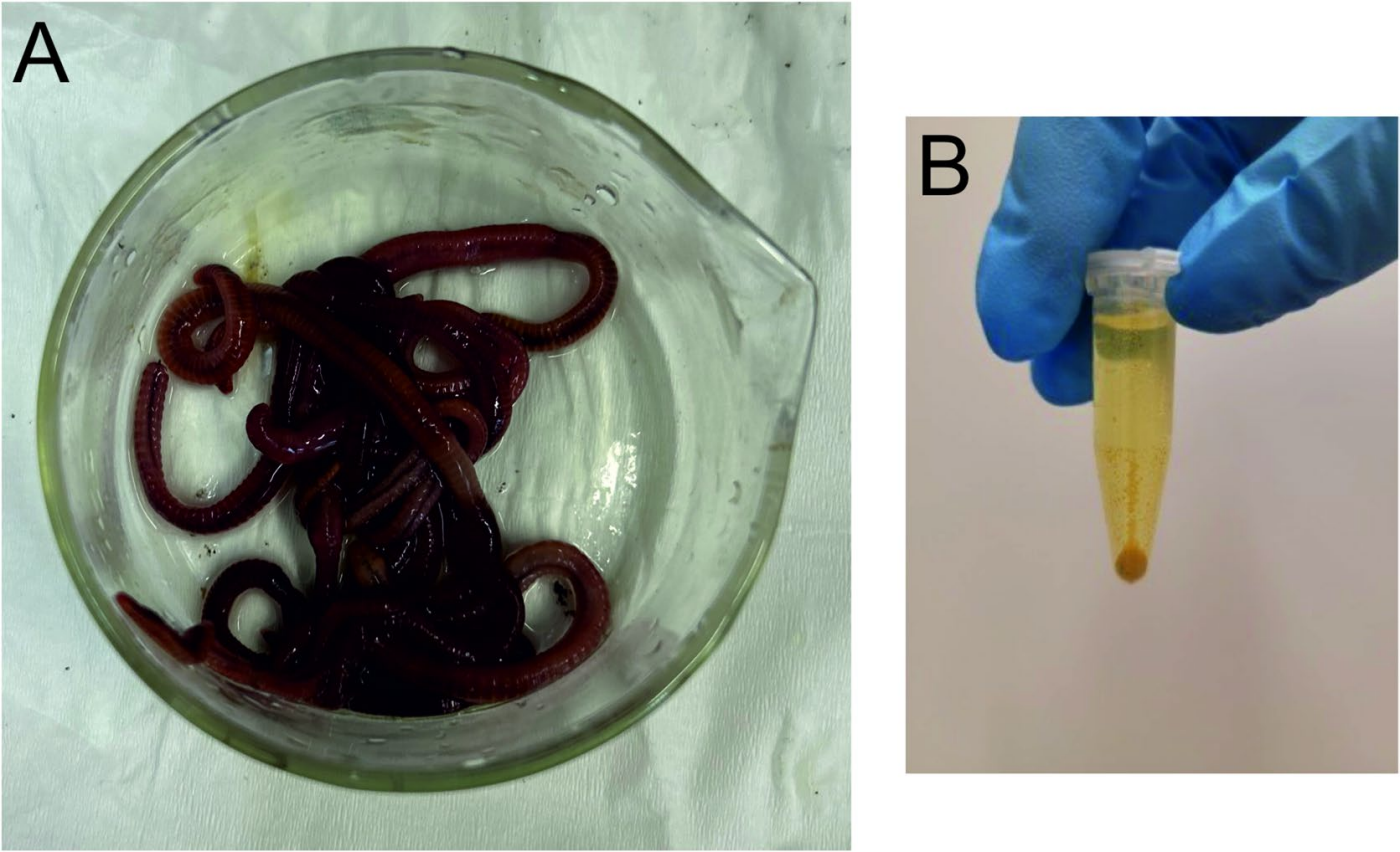
(**A**) *Dendrobaena veneta* earthworms from the laboratory culture; (**B**) isolated coelomic fluid after centrifugation.

Earthworms of the species *D. veneta* were maintained in the laboratory culture of the Department of Immunobiology, Maria Curie-Skłodowska University in Lublin (Poland). The invertebrates were kept in containers filled with compost soil at approx. 20℃ in the dark. They were fed with boiled vegetables and green tea leaves twice a week. Adult earthworms were selected for the experiments (Fig. 1). Earthworms were taken out from the containers and transferred into new ones filled with cellulose for cleaning their guts for 24 h. Subsequently, they were rinsed with barren water and drained on cellucotton. Coelomic fluid (CF) was collected by electrical stimulation (4.5 V) from groups of 10 individuals. The earthworms survived the electric shock and after the treatment were transferred to breeding containers, where after two weeks they regained the proper physiological parameters. CF with coelomocytes was gathered in 0.9% NaCl (1500 µL per group). Then, crude CF was centrifuged at 6,000×g for 10 min at 4℃. The supernatant collected from all Eppendorf tubes was sterilised by filtration through 0.22 μm Millipore filters. The cell-free CF was heated for 10 min at 70℃. Next, it 10 mL of CF was transferred to a cellulose membrane bag with cut-off points of 6–8 kDa. Samples were dialysed against water for 24 h at 4℃. The fraction obtained after the dialysis was transferred into Eppendorf tubes (1 mL), lyophilised, and stored at 20℃. The Bradford assay (Bio-Rad) was used to estimate the protein concentration^35^.

### Preparation of *Candida albicans* cell cultures treated with Venetin-1 and AmB

A clinical isolate of a wild-type strain of *C. albicans* from a patient’s oral mucosa, kindly provided by Prof. A. Kędzia from the Department of Oral Microbiology, Medical University of Gdańsk (Poland), was used for microbiological analyses. Fungal cultivation was initially carried out in Sabouraud liquid medium at a temperature of 28 °C for 24 h. After this time, 30 µl of *C. albicans* culture (1.9-2.0 × 10^7^ CFU, in the logarithmic growth phase) was added to 150 µl of YPD-poor medium (0.1% yeast extract, 0.2% glucose, 0.05% peptone). The samples were supplemented with streptomycin sulphate (Sigma) at a final concentration of 0.17 mg⋅mL^-1^ and the Venetin-1 complex or amphotericin B (Sigma, in 0.2% DMSO). The final protein concentrations of Venetin-1 were 25, 50, and 100 µg⋅mL^-1^, and the concentrations of AmB were 0.125, 0.25, and 0.5 µg⋅mL^-1^. Two control samples without any addition of the preparations or with 0.2% DMSO were prepared in parallel. The final volume of the cell cultures was 250 µL. The samples were incubated at 37 °C for 24 h, with shaking at 700 rpm.

### Analysis of the optical density of * C. albicans* suspensions

To determine the effect of Venetin-1 and AmB on the optical density (OD) of *C. albicans* cultures, the experiments were conducted in liquid cultures. 100 µl of Venetin-1 and AmB were added to 100 µl of yeast suspension in YPD poor medium (10^5^ CFU) to achieve the final protein concentrations of 25, 50, and 100 µg mL^− 1^ for Venetin-1 and final AmB antibiotic concentrations of 0.125, 0.25, and 0.5 µg mL^− 1^. Control cultures with 100 µl of sterile water were prepared simultaneously. Cultures were carried out in 96-well round-bottom plates (Nest Scientific Biotechnology, China) for 24 h at a temperature of 37 °C, with shaking at 600 rpm. OD measurements were performed at a wavelength of 600 nm using a SPECTROstar Nano spectrophotometer (BMG Labtech, Germany) equipped with MARS Data Analysis software. The experiment was performed in triplicate.

### Flow cytometry analysis of * C. albicans* viability

To determine the viability of *C. albicans* control cells and cells incubated with Venetin-1 and amphotericin B at various concentrations, the ViaCount reagent (Luminex) and flow cytometry analysis were used. The ViaCount reagent distinguishes viable and non-viable (apoptotic and dead) cells based on the differential permeability of two DNA-binding dyes. 20 µL of yeast cell suspension were mixed with 780 µL of ViaCount and incubated for 5 min in the dark at room temperature. Flow cytometry analysis was conducted using a Guava easyCyte flow cytometer (Luminex). Emissions were recorded using yellow and green channels. The threshold was set at 68 on the Forward Scatter. 5,000 cells were counted per each sample. Results were analysed using GuavaSoft Software and the EasyFit tool^17^.

### Scanning electron microscopy (SEM) analysis

*Candida albicans* yeast cells from the control and tested cultures were centrifuged (6,000×g, 10 min) and fixed with 4% glutaraldehyde (10 ml of 0.1 M phosphate buffer pH = 7, 10 ml of 8% glutaraldehyde, 200 mg of saccharose) for 2 h at room temperature. Next, the samples were washed with phosphate buffer (pH 7.0) and incubated with a 1.5% water solution of OsO_4_ for 30 min. The cells were then dehydrated in increasing acetone solution concentrations (15%, 30%, 50%, 70%, 100%) by centrifugation at 2,500×g for 30 min at room temperature. The cell suspensions were transferred onto SEM stages with carbon discs and dried in a desiccator for 24 h at room temperature in the presence of silica gel beads. The samples were sputtered with gold using a K550X sputter coater (Quorum Technologies) and imaged using a Vega 3 scanning electron microscope (Tescan) with a 30 kV electron beam voltage and 15,000x magnification^12^.

### Identification of apoptosis and necrosis

To identify apoptosis and necrosis of wild-type *C. albicans* yeast cells after exposure to Venetin-1 and AmB, the cells were stained with a mixture of fluorochromes: 0.15 mg mL^-1^ water solution of propidium iodide (PI) (Sigma) and 0.08 mg mL^-1^ water solution of Hoechst 33342 (Sigma). The staining mixture was added to the yeast suspension in a 1:1 ratio and incubated for 5 min at 37 °C in the dark. Microscopic analysis was performed using a LSM 5 Pa laser confocal microscope (Carl Zeiss, Jena, Germany). Cells were observed at an excitation wavelength of λ = 488 nm with an emission wavelength of λ = 617 nm for PI and at an excitation wavelength of λ = 345 nm with an emission wavelength of λ = 485 nm for Hoechst 33342. Cells undergoing apoptosis were characterised by blue fluorescence of fragmented nuclear material, while cells with red-pink fluorescence were interpreted as necrotic^36^.

### Cell wall analysis using fluorescence microscopy

####  Calcofluor white staining

Calcofluor white (Fluka) is a fluorochrome that binds to chitin in the fungal cell wall. *C. albicans* cell suspensions were incubated with a 30% aqueous solution of Calcofluor white in a 1:1 ratio for 10 min at room temperature in the dark. Then, 2 µL of the suspension was transferred onto a microscope slide and observed under a LSM 5 Pascal confocal microscope (Carl Zeiss, Jena, Germany). Images were obtained using an excitation wavelength of λ = 440 nm and an emission wavelength of λ = 510 nm^17^.

####  Congo red staining

Congo red (Sigma-Aldrich) was used to visualise β-1,4 glucans of the *C. albicans* cell wall. The yeast cell suspension was mixed with a 2% water solution of Congo red in a 1:1 ratio and incubated for 3 min in the dark at room temperature. 2 µL of the preparation was transferred onto a slide and observed under an excitation wavelength λ = 543 nm with the use of an LSM 5 Pascal confocal laser scanning microscope (Carl Zeiss, Jena, Germany) with the magnification of 1000×^17^.

###  Cell wall analysis using flow cytometry

Flow cytometric analysis of the cell wall was performed to quantify unmasked cell wall components, such as β-glucans and chitin, using Fluorescence-Activated Cell Sorting (FACS). Control cells and cells treated with Venetin-1 (100 µg mL^− 1^) and AmB (0.5 µg mL^− 1^) were centrifuged and washed twice with PBS (4,000×g, 5 min). To label exposed chitin, we used a modified protocol developed by Malavia et al.^37^, based on the use of wheat germ agglutinin conjugated with FITC (WGA-FITC) (Sigma-Aldrich). Yeast cells were suspended in a solution of WGA-FITC in PBS (50 µg mL^− 1^) to A600 = 1 and incubated at room temperature for 1 h. Then, the cells were washed with PBS (4,000×g, 5 min), fixed for 15 min in 38% formaldehyde, pelleted, washed, and resuspended in PBS. Unmasked β-glucans were stained according to the modified protocol described by Wagener et al.^38^. Washed *C. albicans* cells were resuspended with 5 µg mL^− 1^ Fc–hDectin-1 (Invivogen) to A600 = 1 and incubated at room temperature for 1 h. Next, the cells were incubated with 1:250 Alexa Fluor 448-conjugated anti-human IgG Fc antibodies (Thermo Fisher, USA) for 1 h on ice. Afterwards, the cells were pelleted, PBS-washed, and fixed (15 min, 38% formaldehyde). For FACS analysis, the cell suspensions were diluted in 500 µl of PBS. The analysis was performed using a Guava easyCyte (EC8) SL system flow cytometer (Luminex, USA) equipped with InCyte software. The measurement of each sample was performed three times, with 5,000 collected events. Gates were set around the cell population using forward and side scatter channels. The fluorescence signal was obtained using a blue laser with a wavelength of 488 nm and the GreenB- channel^39^.

### FTIR analysis of *C. albicans* cells

FTIR spectroscopic analysis is widely used in the study of biological systems. Infrared spectroscopy examines the absorption of radiation associated with the excitation of vibrational levels of molecules, which facilitates the observation of changes in the region of chemical bonds. To investigate changes in *C. albicans* cells after treatment with Venetin-1 and AmB, FTIR spectra were collected from the prepared samples. Yeast cells were rinsed in water and centrifuged (4,000×g 10 min), next, the cells were frozen and lyophilised. The studies were conducted using the ATR (Attenuated Total Reflection) technique directly from the surface of the lyophilised samples^40^. An ALPHA II spectrometer equipped with a single-reflection monolithic diamond crystal (Bruker Optics GmbH & Co. KG, Ettlingen, Germany) was used for the studies. Measurements were performed at room temperature in the spectral range of 4,000–400 cm^− 1^ at a resolution of 4 cm^− 1^ and with a 32-scan rate for the sample and the background.

###  Young’s modulus analysis of *C. albicans* cells using atomic force microscopy (AFM)

Young’s modulus is defined as “stress/strain” and reflects the stiffness of a material. It is used to assess cytomechanical changes in the cell wall of live microorganisms. *C. albicans* control and tested cells were washed twice with PBS (4,000×g, 5 min) and resuspended in sterile water. 2 µl of each suspension were applied to mica discs. To estimate the effect of Venetin-1 and AmB on yeast cell wall elasticity, measurements were performed using the Peak Force QNM technique with a NSG30 probe (NT-MDT) with a force constant of 38 N/m, a resonant frequency of 310 kHz, and a tip curvature radius of 10 nm. The scanning parameters were as follows: scan size: 1 μm x 1 μm with a scanning frequency of 1 Hz. A MultiMode™8- NanoScope^®^ V (Bruker-Veeco) microscope equipped with Bruker Research Nanoscope 8.15 and Bruker Nanoscope Analysis 1.40 software was used. The data presented are means of three independent measurements with standard deviation (± SD).

###  Proteomic analysis

#### Preparation of proteomic samples

The experimental design included one control sample and three samples of *C. albicans* cells treated with AmB at final concentrations of 0.125, 0.25, and 0.50 µg mL^− 1^ and Venetin-1 at final concentrations of 25, 50, and 100 µg mL^− 1^. Lyophilised *C. albicans* cells were suspended in lysis buffer (2% SDS, 100 mM TRIS, 10 mM DTT) and heated in a thermoblock at 95 °C for 10 min. The samples were subsequently sonicated using a QSonica Q700MPXC sonicator with Micro Plate Horn system (amplitude 50; total sonication time 5 min, consisting of cycles of 5 s on and 5 s off). Protein digestion was performed according to the standard filter-aided sample preparation (FASP) protocol routinely used in our laboratory^41^. Trypsin was used as the proteolytic enzyme, and digestion was carried out overnight at 37 °C using a 10 kDa molecular weight cut-off membrane. After digestion, peptides were purified using homemade C18 StageTips according to the Rappsilber method^42^. Following purification, 10 µg of peptides was collected and diluted to a final volume of 30 µL for LC–MS/MS analysis.

#### LC–MS/MS analysis

LC–MS/MS analyses were performed using a ZenoTOF™ 7600 mass spectrometer (SCIEX) coupled to an M5 MicroLC system (SCIEX) equipped with a PAL3 autosampler. Data acquisition was controlled using SCIEX OS software version 3.4.0. Peptides were analysed in positive ion mode using an OptiFlow 1–50 µL Micro/MicroCal ion source. The ion source parameters were set as follows: curtain gas 35, CAD gas 7, ion source gas 1 at 20 psi, ion source gas 2 at 40 psi, and source temperature at 400 °C. The spray voltage was set to 5000 V. Column temperature was maintained at 40 °C.

#### Liquid chromatography

Peptide separation was performed using a trap-and-elute configuration. Peptides were first loaded onto a trap column (HALO C18, particle size 5 μm, 90 μm × 15 mm) at a flow rate of 50 µL/min with 5% solvent B. The analytical separation was carried out at a flow rate of 10 µL/min using solvent A (water with 0.1% formic acid) and solvent B (acetonitrile with 0.1% formic acid). A 28-min gradient was applied as follows: 5–15% B in 1 min, 15–20% B over 7 min, 20–30% B over 8 min, 30–45% B over 4 min, followed by a wash at 95% B and re-equilibration to initial conditions. The injection volume was 5 µL. Samples were maintained at 8 °C in the autosampler.

#### Mass spectrometry – IDA acquisition

Information-dependent acquisition (IDA) was performed with a TOF-MS survey scan range of m/z 300–1600 and a TOF-MS/MS scan range of m/z 50–2000. The accumulation time was set to 100 ms for MS scans and 12 ms for MS/MS scans. A maximum of 40 precursor ions per cycle were selected for fragmentation with an intensity threshold of 100 counts/s. Dynamic collision energy was applied using charge-state-dependent equations, with collision energies ranging from 5 to 80 V. Only precursor ions with charge states from 2 + to 5 + were selected. Zeno pulsing was enabled for MS/MS acquisition.

#### Proteomic data processing

Raw data files were acquired in the WIFF2 format and processed using ProteinPilot software. A spectral library was generated from IDA runs of control and AmB–treated samples. Protein identification was performed against the UniProt *C. albicans* database (download date: January 2026). Each sample was analysed in three technical replicates. Relative quantitative analysis was carried out using the SWATH-MS approach. Statistical significance was determined using a q-value threshold of < 0.05. Differentially abundant proteins were defined using fold-change thresholds of ≥ 1.5 for upregulated proteins and ≤ 0.7 for downregulated proteins. Each treated group was compared with the control samples. Protein –protein interaction analysis was performed using STRING (latest available version)^43^, and network visualization was conducted using Cytoscape (latest available version)^43^.

Quantitative proteomic data were processed and statistically analysed using Perseus software following established workflows for label-free quantitative proteomics^44^. Data preprocessing included filtering, normalisation and permutation-based statistical testing with false discovery rate (FDR) control. Principal component analysis (PCA) was applied to assess global variance and sample clustering, and correlation analyses were performed to evaluate reproducibility between biological replicates and overall dataset consistency. Functional enrichment analysis of differentially regulated proteins was conducted using STRING (version 2.1.6.0, Max Planck Institute of Biochemistry) to identify overrepresented Gene Ontology terms and pathway annotations derived from KEGG and Reactome databases^43,45,46^.

###  Electrokinetic analysis

Electrophoretic mobility, conductivity, and zeta potential were measured for 10⁻³ mol/dm³ KNO₃ and KCl solutions using a Zetasizer Nano-ZS instrument (Malvern). Since the κa value was around 150, Smoluchowski’s equation was used to calculate the zeta potential. Venetin-1 or amphotericin B was added to the solution at a concentration of 100 ppm. The mixture was then dispersed using an ultrasonic probe (Sonicator XL 2020, Misonix). Subsequently, the suspension was transferred into 125 mL flasks, and the pH was adjusted to values between 2 and 11 using 0.1 mol/dm³ HNO₃ and KOH solutions. For each sample, six measurements of electrophoretic mobility, conductivity, and zeta potential were taken. The results are shown in the diagrams below. Potassium nitrate (KNO₃) and potassium chloride (KCl) were used as an electrolyte for the zeta potential measurements. They were selected as background electrolytes due to their chemical inertness toward Venetin-1/AmB, the high ionic mobility of K⁺ ions, and their ability to provide a well-defined ionic strength. The use of both electrolytes allowed assessment of potential specific anion effects (Cl⁻ vs. NO₃⁻) on the electrokinetic properties of the studied systems.

### Statistical analysis

The results obtained in the study are presented as the mean of at least three independent repetitions, taking into account the standard deviation (± SD). The Shapiro–Wilk test was used to check the type of data distribution, and the Levene test was employed to determine the homogeneity of variance. The post-hoc Tukey HSD test and one-way ANOVA were used to examine the level of significance. Statistical significance was assumed at * *p* < 0.05, ** *p* < 0.01, and *** *p* < 0.001. The analysis was performed using Statistica (Tibco Software Inc., USA).

The list of reagents with full names and abbreviations is available in the supplementary materials as Supplementary Table S1.

## Results

### Optical density analysis

**Fig. 2:**
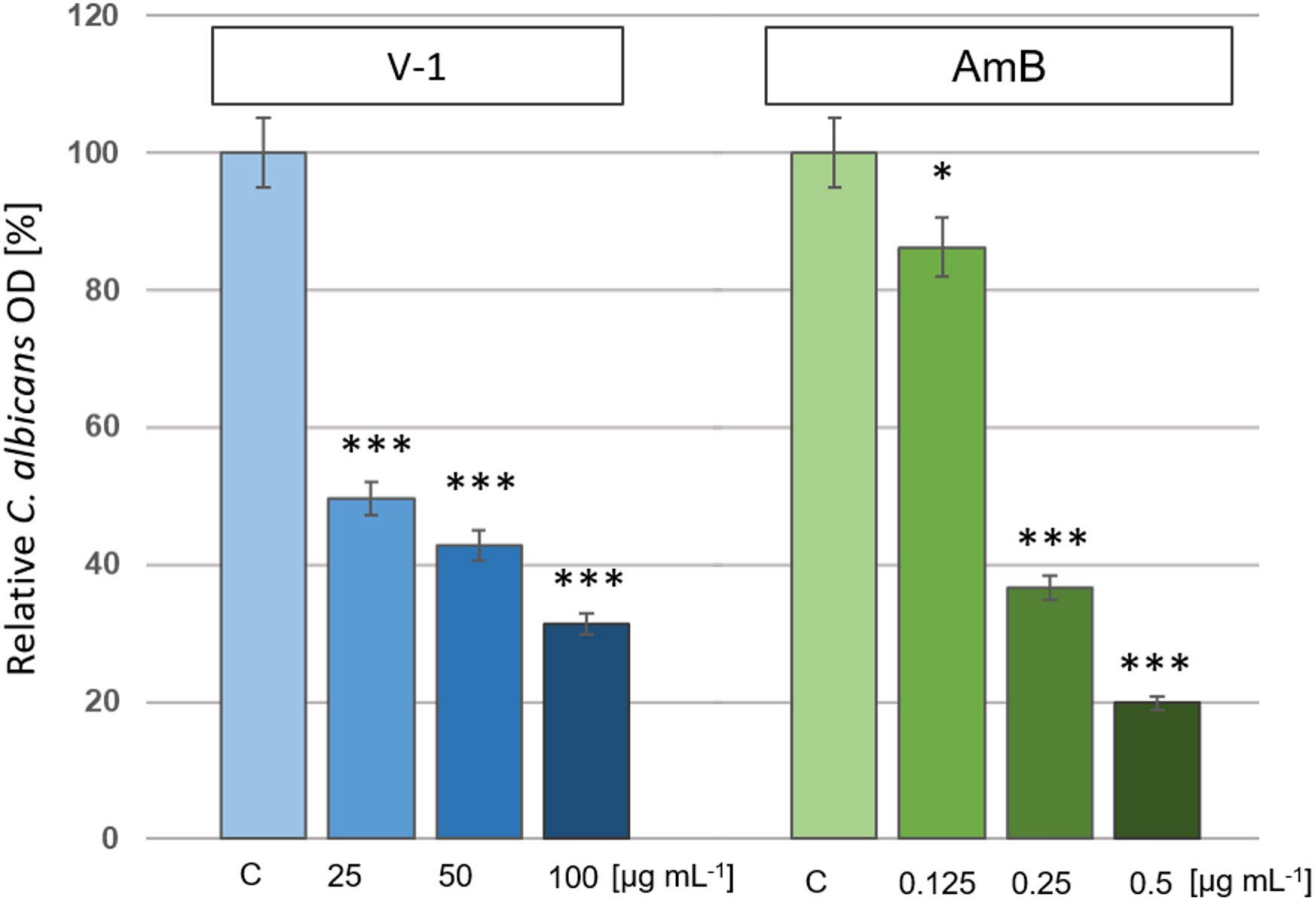
Relative *C. albicans* optical density after treatment with Venetin^-1^ and amphotericin B at different concentrations for 24 h. C – control; statistical significance compared to the control *p ≤ 0.05; ***p ≤ 0.001. The graphs show the results from three independent experiments.

The spectrophotometric measurements showed a decrease in the relative optical density of *C. albicans* after the treatment with both Venetin-1 and amphotericin B (Fig. 2). The most pronounced changes were observed in the case of cultures incubated with AmB, where the largest decrease in OD, by 80% compared to the control culture, occurred after the antibiotic treatment at a concentration of 0.5 µg mL^− 1^. The OD was lower by over 60% at an AmB concentration of 0.25 µg mL^− 1^ and by 14% at 0.125 µg mL^− 1^. After incubation of yeast cultures with Venetin-1, the decrease in optical density was also dependent on the concentration used. The relative OD of *C. albicans* after the Venetin-1 treatment was 31% at the highest concentration of 100 µg mL^− 1^, 43% at 50 µg mL^− 1^, and 50% at 25 µg mL^− 1^. All observed changes were statistically significant.

### Flow cytometry analysis of *C. albicans* viability

**Fig. 3:**
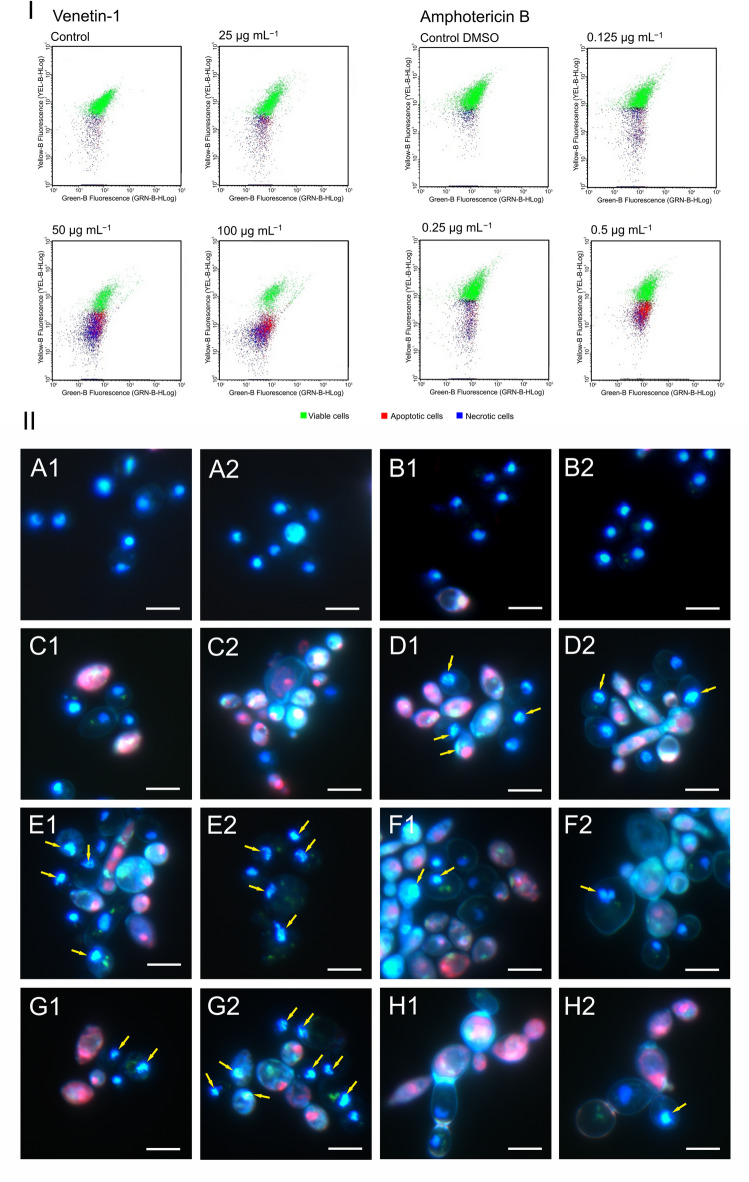
Effect of Venetin-1 (V-1) and amphotericin B on the survival of *C. albicans* cells and on the type of induced cell death after 24 h incubation. (**I**) - Flow cytometric analysis using the ViaCount reagent demonstrates the proportion of living cells, cells undergoing apoptosis, and those undergoing necrosis in the culture. Presented data are representative of three independent experiments. (**II**) Fluorescence microscopic analysis using propidium iodide and Hoechst 33,342 staining: A1, A2 - control culture cells; B1, B2 – DMSO control culture cells; C1, C2 - cells after incubation with V-1 at 25 µg mL^− 1^; D1, D2 - cells after incubation with AmB at 0.125 µg mL^− 1^; E1, E2 - with V-1 at 50 µg mL^− 1^; F1, F2 – with AmB at 0.25 µg mL^− 1^; G1, G2 - with V-1 at 100 µg mL^− 1^; H1, H2 - with AmB at 0.5 µg mL^− 1^. Yellow arrows indicate cells undergoing the apoptotic process with fragmented genetic material fluorescing intense light blue. Cells undergoing necrosis are fluorescing pink. The scale bars represent 5 μm.

The flow cytometric analysis facilitated the quantification of the percentage of viable, apoptotic, and necrotic cells in the control and tested *C. albicans* cell cultures (Table 1; Fig. 3I). It was shown that the proportion of viable cells in the *C. albicans* control culture and the DMSO control was 70.66% and 80.82%, respectively. After incubation of cells with Venetin-1 at a concentration of 25 µg ml^− 1^, the percentage of viable cells in the culture dropped to almost 58%, and was slightly over 31% at a concentration of 50 µg ml^− 1^. In both cultures, necrotic cells predominated in the population of non-viable cells. In the case of the AmB-treated cultures, the percentage of viable cells after the exposure to the concentrations of 0.125 µg ml^− 1^ and 0.25 µg ml^− 1^ was significantly different from that in the control culture, as it reached 64.84 and 66.22%, respectively. In these cultures, necrotic cells similarly constituted the majority of dead cells. The Venetin-1 preparation at the highest concentration (100 µg ml^− 1^) caused a greater decrease in the viability of yeast cells: 32.10% were apoptotic cells and 39.58% were necrotic (71.68% in total), compared to AmB used at the highest concentration (0.5 µg ml^− 1^), where 49.24% of cells were non-viable (apoptotic cells − 31.9%, necrotic cells − 17.34%).

**Table 1:** Percentage of live and dead *C. albicans* cells in control cultures and cultures incubated with Venetin-1 and amphotericin B at various concentrations for 24 h. Analysis performed using ViaCount reagent and flow cytometry. Results are means of three independent measurements with standard deviation (± SD).

Concentration [µg ml^− 1^]	Viable cells [%]	Apoptotic cells [%]	Necrotic cells [%]
Control	70.66 (± 2.67)^c^	4.74 (± 1.07)^a^	24.6 (± 3.74)^a^
Venetin-1 25	57.74 (± 6.32)^b^	11.34 (± 1.59)^b^	30.92 (± 7.91)^a^
Venetin-1 50	31.22 (± 5.23)^a^	23.28 (± 3.33)^c^	45.50 (± 8.56)^b^
Venetin-1 100	28.32 (± 1.46)^a^	32.10 (± 1.83)^d^	39.58 (± 1.13)^b^
Control DMSO	80.82 (± 5.50)^c^	1.98 (± 0.61)^ab^	17.20 (± 4.98)^a^
AmB 0.125	64.84 (± 2.93)^c^	6.58 (± 0.73)^ab^	28.58 (± 3.62)^a^
AmB 0.25	66.22 (± 6.25)^c^	8.6 (± 0.7)^ab^	25.18 (± 6.91)^a^
AmB 0.5	50.6 (± 4.28)^b^	31.9 (± 3.06)^d^	17.34 (± 7.26)^a^

Means followed by the same lowercase letter in the column do not differ statistically from each other as determined by Tukey’s test at *p* < 0.05.

Changes in the number of viable *C. albicans* cells following V-1 treatment (at all concentrations used) were statistically significant compared to the control. In the case of AmB, only incubation with the highest antibiotic concentration (0.5 µg ml^− 1^) resulted in a statistically significant reduction in the number of viable cells compared to the DMSO control. Incubation with V-1 resulted in a greater decrease in yeast cells viability than incubation with AmB under the experimental conditions used.

### Identification of apoptosis and necrosis

*C. albicans* cells treated with Venetin-1 and AmB were stained with a mixture of Hoechst33342 and propidium iodide to identify the cell death pathway. Control cells with the addition of water and the DMSO solution (used as solvents for active antifungal compounds) exhibited dark blue luminescence of regular cell nuclei, as visualised in images Fig. 3 II A, B1, B2, and B2. Following the Venetin-1 treatment at a concentration of 25 µg mL^-1^, necrotic cells fluorescing pink were observed alongside blue cells (Fig. 3 II C1, C2). Following the Venetin- 1 treatment at concentrations of 50 µg mL^-1^ and 100 µg mL^-1^, both necrotic and apoptotic cells were detected, as indicated by yellow arrows in Fig. 3 II E1, E2, G1, and G2. The cell nuclei in these images are irregular, fragmented, and fluoresce intensely blue-white. After incubation of *C. albicans* cells with AmB at concentrations of 0.125, 0.250, and 0.5 µg mL^-1^, the microscopic analysis revealed both necrotic cells with pink fluorescence and apoptotic cells with white-blue fluorescent nuclei at various stages of apoptosis, as indicated by yellow arrows in Fig. 3 II D1, D2, F1, and H2. In image F2, corresponding to treatment with AmB at a concentration of 0.25 µg mL^-1^, the fragmentation stage of nuclear material is clearly visible.

### SEM analysis of *C. albicans* cells

**Fig. 4:**
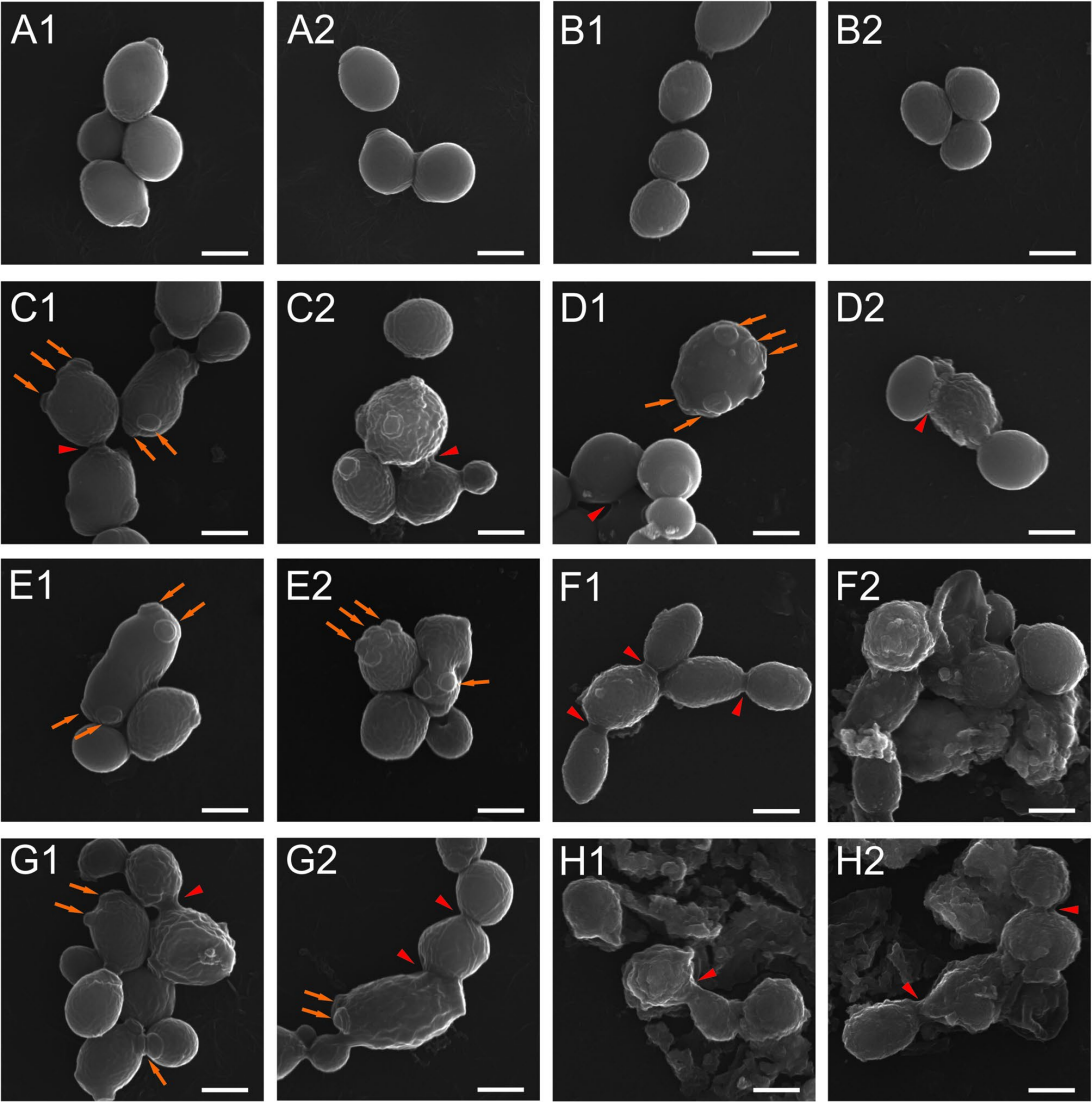
SEM analysis of *C. albicans* cells after incubation with Venetin-1 (V-1) and AmB at different concentrations for 24 h: (**A1**, **A2**) control culture cells; (**B1**, **B2**) DMSO control culture cells; (**C1**, **C2**) cells after incubation with V-1 at 25 µg mL^− 1^; (**D1**, **D2**) cells after incubation with AmB at 0.125 µg mL^− 1,^ (**E1**, **E2**) with V-1 at 50 µg mL^− 1^; (**F1**, **F2**) with AmB at 0.25 µg mL^− 1^; (**G1**, **G2**) - with V-1 at 100 µg mL^− 1^; (**H1**, **H2**) - with AmB at 0.5 µg mL^− 1^. Orange arrows indicate cells with numerous division scars; red arrowheads indicate unseparated mature cells. The scale bars represent 2 μm.

The SEM images of *C. albicans* control culture cells and cells treated with Venetin-1 and amphotericin B showed the fungal cell morphology. Control cells were characterised by a regular shape and a smooth cell wall (Fig. 4A1, A2, B1, B2). Cells incubated with the DMSO solution, used in the experiment as a solvent for AmB, showed no morphological differences compared to cells incubated with water as a control solvent for Venetin-1. The analysis of cells incubated with Venetin-1 at concentrations of 25 µg mL^-1^ and 50 µg mL^-1^ showed cell aggregation, the presence of cells with multiple scars at one pole (indicated by orange arrows), deformed cells, and cells with a wrinkled cell wall (Fig. 4C1, C2, E1, E2). After the application of Venetin-1 at a concentration of 100 µg mL^-1^, chains of connected cells, unseparated cells with a strongly folded cell wall, and cells with a clear loss of cell wall integrity were observed (Fig. 4G1, G2). The locations of unseparated mature cells are marked with red arrowheads in Fig. 4C2 and G2. After incubation with AmB at a concentration of 0.125 µg mL^-1^, morphological changes were similar to those observed after the treatment with the lowest concentration of Venetin-1. Cells with numerous division scars (Fig. 4D1, indicated by orange arrows) and loss of wall integrity in a clump of cells were observed (Fig. 4D2). After the application of AmB at a concentration of 0.5 µg mL^-1^, cells connected in chains and wall degradation in the multi-cell aggregate could be observed (Fig. 4F1, F2). After the highest concentration of AmB (1.0 µg mL^-1^), the loss of cell shape was most evident, with deformed cells visible as irregular links in the *Candida* cell chain (Fig. 4H1, H2). At each AmB concentration, disturbances in the formation of a functional cell wall were observed; the resulting fusion of mature cells is indicated by red arrowheads (Fig. 4D2, F1, H1, H2).

### Cell wall analysis using fluorescence microscopy

#### Staining of chitin with Calcofluor white

**Fig. 5:**
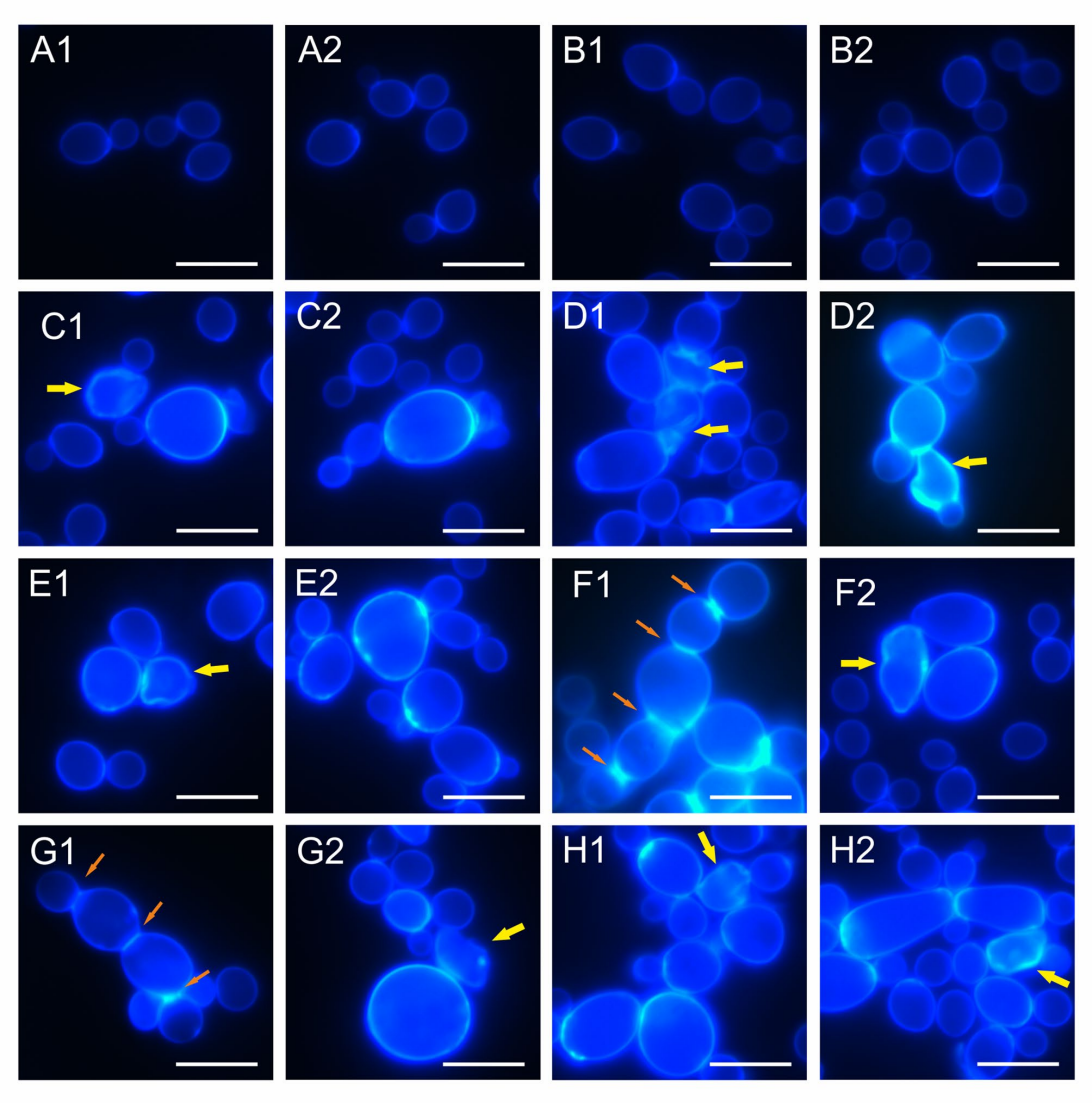
Morphological changes and total chitin staining using Calcofluor white of *C. albicans* cells after incubation with Venetin-1 (V-1) and AmB at different concentrations for 24 h observed under the CLSM microscope: (**A1**, **A2**) control culture cells; (**B1**, **B2**) DMSO control culture cells; (**C1**, **C2**) cells after incubation with V-1 at 25 µg mL^− 1^; (**D1**, **D2**) cells after incubation with AmB at 0.125 µg mL^− 1^; (**E1**, **E2**) with V-1 at 50 µg mL^− 1^; (**F1**, **F2**) with AmB at 0.25 µg mL^− 1^; (**G1**, **G2**) with V-1 at 100 µg mL^− 1^; (**H1**, **H2**) with AmB at 0.5 µg mL^− 1^. Yellow arrows indicate cells with an irregular shape and loss of cell wall integrity; orange arrows indicate chitin-rich junctions between yeast cells. The scale bars represent 5 μm.

After staining of *C. albicans* cells with the fluorochrome Calcofluor white, the control culture cells for Venetin-1 with water and AmB with DMSO exhibited very similar morphology. They emitted blue-white fluorescence with a distinct cell wall outline, i.e. the site where the dye binds to chitin (Fig. 5A1, A2, B1, B2). After incubation of yeast cells with Venetin-1 at concentrations of 25 and 50 µg mL^-1^, large cells with a strongly luminescent blue-white cell wall (Fig. 5C1, C2, E2) and cells of irregular shape that had lost cell wall integrity (marked with yellow arrows in Fig.5C1, E1) were observed. After treatment with Venetin-1 at a concentration of 100 µg mL^-1^, loss of normal cell shape was also observed, with some cells being several times larger than those observed in the control (Fig. 5G2). In addition, chains of several interconnected cells were visualised. Their luminous, chitin-rich junctions are marked with orange arrows in Fig. 5G1. After incubation of *C. albicans* with AmB at concentrations of 0.125 and 0.25 µg mL^-1^, loss of regular shape was frequently observed (Fig. 5D1, D2, F2, marked with yellow arrows). In addition, after exposure to amphotericin B at a concentration of 0.25 µg mL^-1^, chains formed by multiple cells were observed in the culture. A white-fluorescent connection between them, with a large amount of accumulated chitin, is marked with orange arrows in Fig. 5F1. After incubation in the presence of AmB at the highest concentration used (0.5 µg mL^-1^), the culture comprised oval and elongated giant cells transforming into pseudohyphae (Fig. 5H1, H2) and cells of normal size but with irregular shape (marked with yellow arrows in Fig. 5H1, H2).

**Fig. 6:**
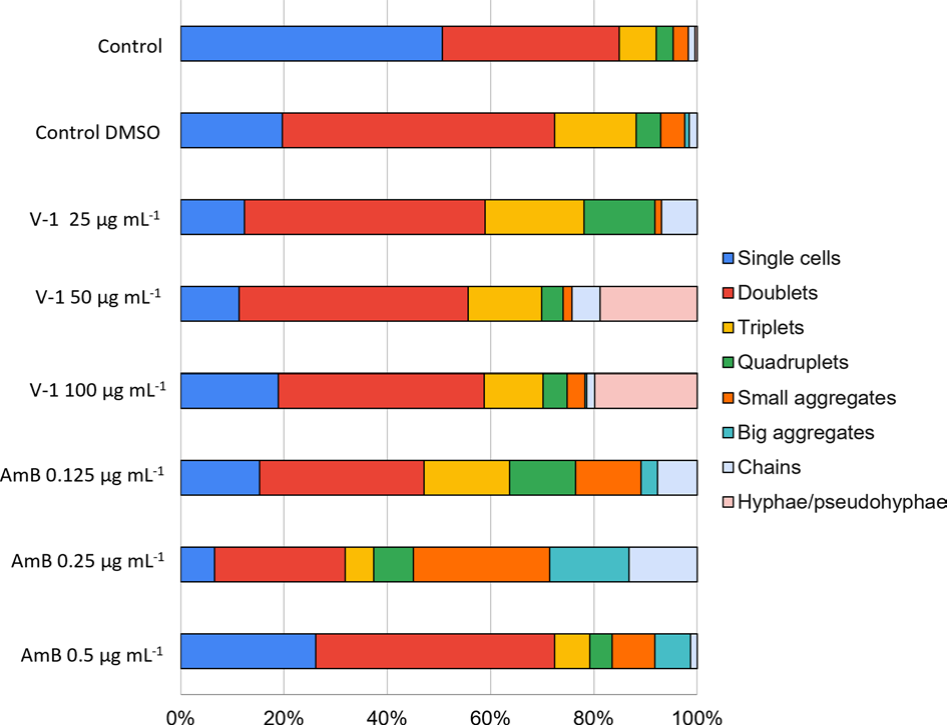
Quantitative representation of *C. albicans* cell aggregates in control cultures and cultures after 24 h of incubation with Venetin-1 and amphotericin B at different concentrations.

After staining with Calcofluor white, quantitative analysis of aggregated *C. albicans* cell forms was performed Fig. 6. The control cultures were dominated by single cells and doublets resulting from normal cell division. After incubation with Venetin-1 at a concentration of 25 µg mL^-1^, additional triplets appeared, constituting 19% and quadruplets – 14% of all forms. After the use of Venetin-1 at concentrations of 50 and 100 µg mL^-1^, approximately 20% of all forms were hyphae and pseudohyphae, which were not observed at the lowest concentration (Fig. 6). After the application of AmB at the lowest concentration of 0.125 µg mL^-1^, the group of triplets, quadruplet and small aggregates constituted each about 15% of the entire culture. AmB at a concentration of 0.25 µg mL^-1^increased the number of small and large cell aggregates and chains. However, AmB concentration of 0.5 µg mL^-1^ caused a decrease in the number of aggregates and an increase in the number of doublets compared to lower concentrations of this antibiotic (Fig. 6).

#### Staining of β- glucans with Congo red

**Fig. 7:**
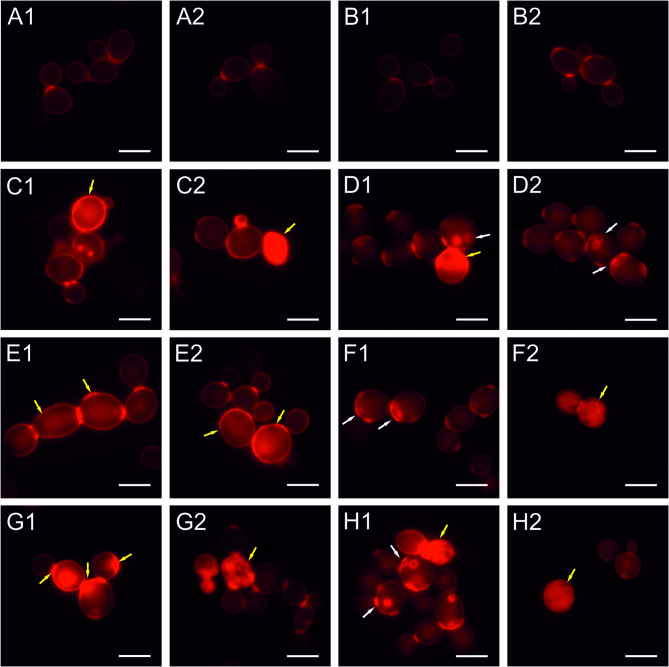
β-glucan staining using Congo Red in *C. albicans cells* after incubation with Venetin- 1 (V-1) and AmB at different concentrations for 24 h observed under the CLSM microscope: (**A1**, **A2**) control culture cells; (**B1**, **B2**) DMSO control culture cells; (**C1**, **C2**) cells after incubation with V-1 at 25 µg mL^− 1^; (**D1**, **D2**) cells after incubation with AmB at 0.125 µg mL^− 1^; (**E1**, **E2**) with V-1 at 50 µg mL^− 1^; (**F1**, **F2**) with AmB at 0.25 µg mL^− 1^; (**G1**, **G2**) with V-1 at 100 µg mL^− 1^; (**H1**, **H2**) with AmB at 0.5 µg mL^− 1^. Yellow arrows indicate cells with strong β-glucan layer fluorescence; white arrows indicate strongly fluorescent post-division scars. The scale bars represent 5 μm.

After staining of *C. albicans* cells with Congo red, the control culture cells were observed to exhibit very weak red fluorescence. Only cell outlines and intercellular junctions, which fluoresced moderately red, were visible (Fig. 7A1, A2, B1, B2). After incubation of yeast cells with Venetin-1 at concentrations of 25 and 50 µg mL^− 1^, the cell surface of some cells fluoresced strongly red, revealing the β-glucan layer (Fig. 7C1, C2, E1, E2, changes indicated by yellow arrows). Additionally, after exposure to Venetin-1 at a concentration of 50 µg mL^− 1^, chains of cells with intensely fluorescent intercellular junctions were observed (Fig. 7E1). After applying the highest concentration of 100 µg mL^− 1^, cells with a regular shape and deformed cells with loss of cell wall integrity exhibited an intensely luminous surface (Fig. 7G1, G2). The intense fluorescence is indicated by yellow arrows in these images. After exposure of the culture to AmB at concentrations of 0.125, 0.25, and 0.5 µg mL^− 1^, stronger red fluorescence of the wall was observed in each of these cases (marked with yellow arrows in Fig. 7D1, F2, H1, H2), along with strongly fluorescent post-division scars (Fig. 7D1, D2, F1, H1, indicated by white arrows). Additionally, intensely luminous walls of deformed and undivided cells were visible (Fig. 7H1).

### Cell wall analysis using flow cytometry

**Fig. 8:**
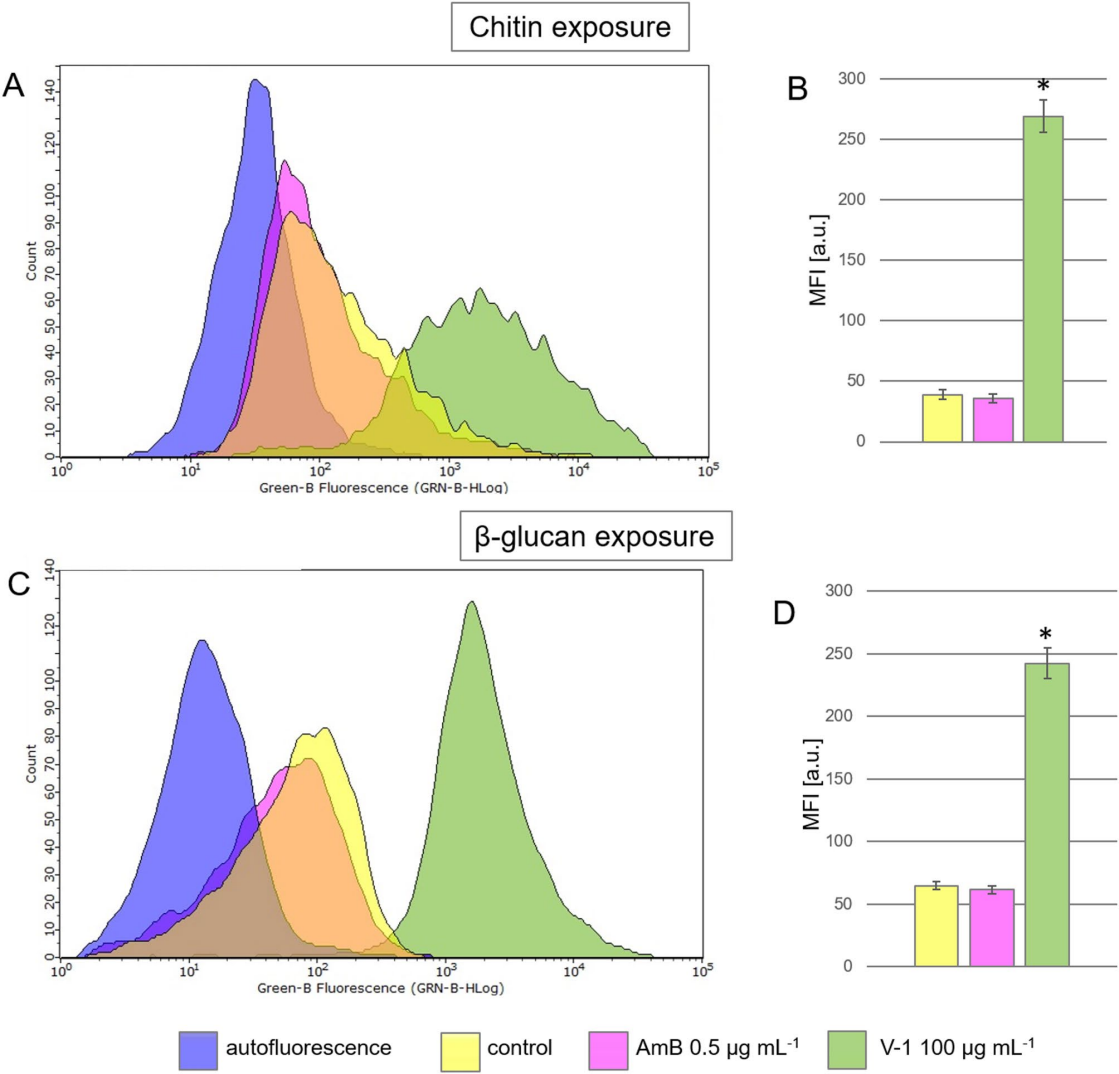
Exposure of cell wall layers in *C. albicans* cells after incubation with Venetin-1 (V- 1) (100 µg mL^−^ 1) and amphotericin B (0.5 µg mL^− 1^) for 1 h measured using a flow cytometer: (**A**) exposure of chitin layer examined after staining of yeast cells with wheat germ agglutinin conjugated with FITC (WGA-FITC); (**B**) mean fluorescence intensity (MFI) of exposed chitin; (**C**) β-glucan exposure after staining of cells with Fc-hDectin-1 and Alexa Fluor 448-conjugated anti-human IgG Fc antibody; (**D**) MFI of exposed β-glucan. Autofluorescence of unstained *C. albicans* cells was subtracted. Presented data are representative of three independent experiments. MFIs were quantified for all three experiments; statistical significance compared to the control ***p ≤ 0.001; a. u. - arbitrary unit.

The flow cytometric analysis of the *C. albicans* cell wall was aimed at assessing whether and to what extent wall components are exposed after incubation of cells with Venetin-1 and AmB. Two elements of the cell wall were stained: chitin with WGA-FITC and β-glucans with FC-Dec1. The fluorescence intensity was quantified by flow cytometry. Statistically significantly higher values for both unmasked chitin and β-glucan were recorded after treatment of *C. albicans* cells with Venetin-1 at a concentration of 100 µg mL^− 1^, compared to 0.5 µg mL^− 1^ AmB (Fig. 8). Incubation of yeast cells with Venetin-1 shifted the green fluorescence to the right both for chitin and β-glucan (Figs. 8A, C). In the case of chitin externalisation, the mean fluorescence intensity (MFI) value indicates a 6.8-fold increase after Venetin-1 treatment (268.78 units (± 9.12)) in comparison to the control cells (39.04 units (± 1.15)) and stability after incubation of yeast cells with AmB (36.09 units (± 3.20)) (Fig. 8B). The MFI for β-glucans also indicated an exposure effect only after Venetin-1 treatment, where its value increased from 64.66 units (± 1.59) in the control culture to 242.43 units (± 3.53) in the tested culture (Fig. 8D). There were no significant changes in β-glucan fluorescence between control *C. albicans* cells and cells exposed to AmB − 61.49 units (± 1.83) (Fig. 8D).

### FTIR analysis

**Fig. 9:**
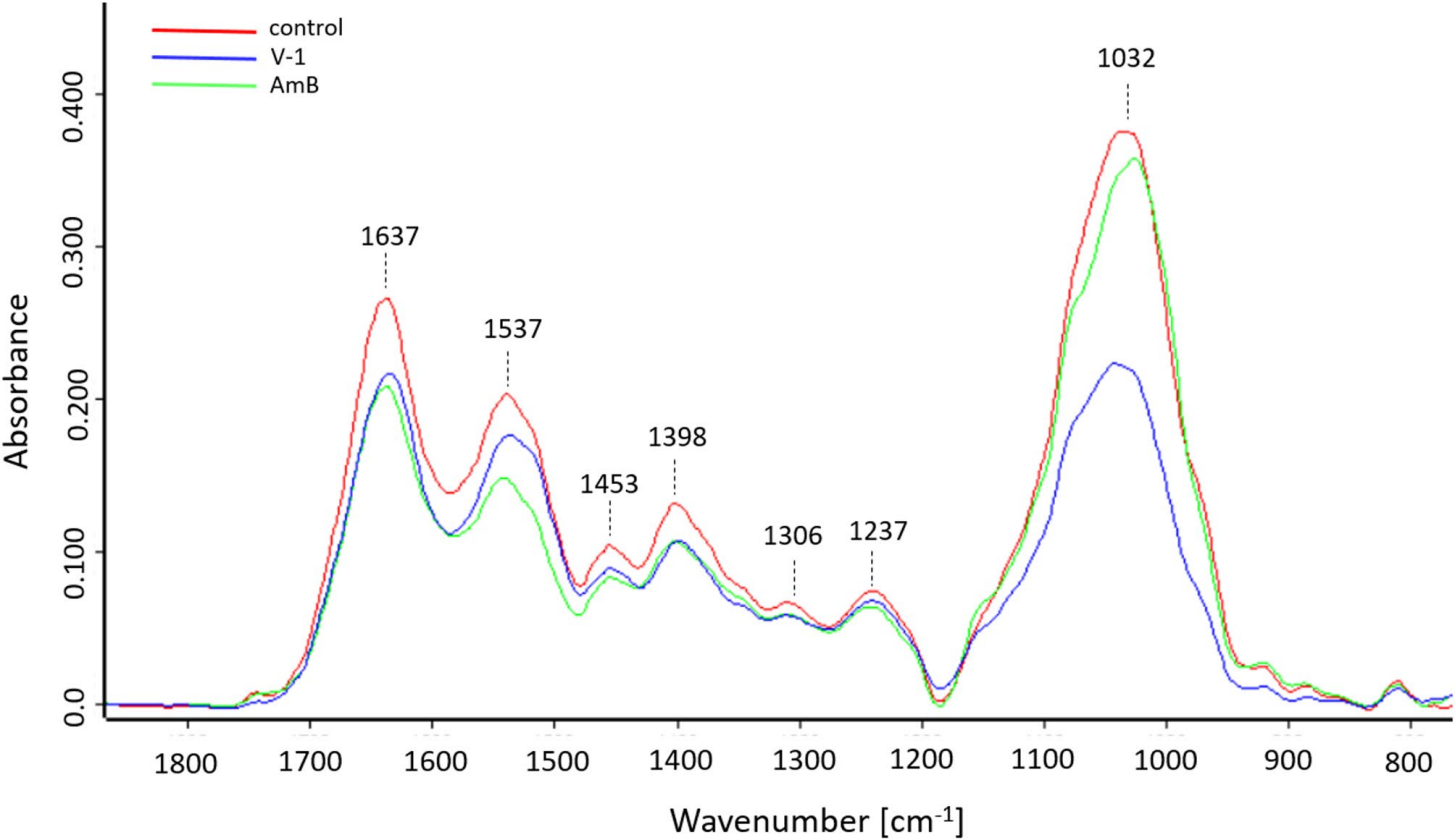
FTIR spectra in the range of 1800 –800 cm^− 1^ of *C. albicans* control cells (red) and cells after incubation with Venetin-1 (100 µg mL^−^^1^) (blue) and amphotericin B (0.5 µg mL^− 1^) (green) for 24 h.

FTIR spectra for *C. albicans* cells treated with Venetin-1 (100 µg mL^− 1^), amphotericin B (0.5 µg mL^− 1^), and untreated control cells were recorded in the spectral range of 4000–400 cm^- 1^. However, during the analysis of the obtained signals, the focus was placed on the region of 1800–800 cm^-1^, where the most pronounced differences in the spectra were observed (Fig. 9). Based on the literature data, selected signals were analysed with assignment of characteristic chemical bonds^47–49^. In the presented spectra, the ranges assigned to the signal characteristic of vibrations corresponding to bonds of individual yeast macromolecules can be distinguished: 1720–1250 cm^− 1^ exhibited peaks corresponding to vibrations characteristic of bonds occurring in proteins, while bands characteristic of polysaccharides were observed in the range of 1250–750 cm^− 1^. The measurements showed a decrease in signals in the tested region after *C. albicans* treatment with both Venetin-1 and AmB. The spectrum of AmB-treated cells was characterised by a more significant reduction in signal intensity than that of Venetin-1 -treated cells at 1637 cm^− 1^ (amide I), 1537 cm^− 1^ (amide II), 1453 cm^− 1^ (lipids), and 1237 cm^− 1^ (phosphomannan). In turn, a greater reduction in the signal intensity of the 1032 cm^− 1^ absorption band, originating from polysaccharides (mannans), was observed in the spectrum of cells exposed to Venetin-1 than those incubated with AmB.

### AFM analysis

The atomic force microscopy measurements of Young’s modulus showed changes in the values of this parameter for *C. albicans* cells after incubation with Venetin-1 (100 µg mL^− 1^) and AmB (0.5 µg mL^− 1^), compared to the control culture. The value of Young’s modulus was 6451.67 MPa (± 627.64) for control cells, 3566.25 MPa (± 258.08) for cells treated with Venetin-1, and 3453.25 MPa (± 148.26) after AmB treatment. A decrease in these values for the treated cells indicates a change in the nanomechanical properties of the cell wall, i.e. an increase in elasticity relative to the control cells.

## Proteomic analysis

Proteomic samples were prepared and processed as described in the Methods section and analysed using SWATH-MS quantitative proteomics. Comparative analysis included *C. albicans* cells exposed to amphotericin B at 0.125, 0.25 and 0.5 µg mL⁻¹ and Venetin-1 at 25, 50 and 100 µg mL⁻¹, alongside corresponding controls.

**Fig. 10:**
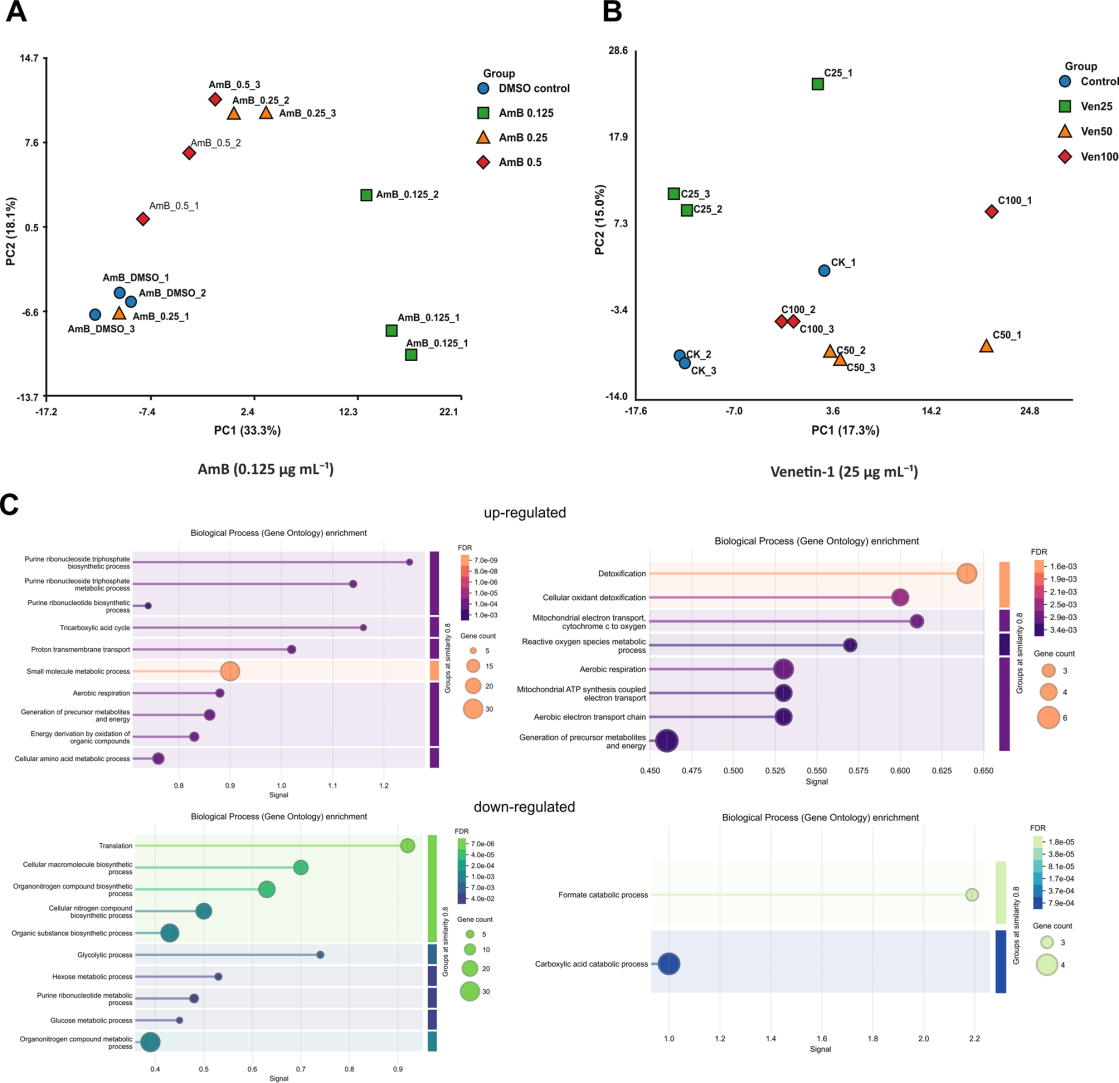
Comparative proteomic analysis of *C. albicans* cells treated with amphotericin B and Venetin-1. (**A**) principal component analysis (PCA) of samples treated with amphotericin B (AmB) at concentrations of 0.125, 0.25 and 0.5 µg mL⁻¹ and corresponding controls. (**B**) PCA of samples treated with Venetin-1 at concentrations of 25, 50 and 100 µg mL⁻¹ and control samples. Each point represents an individual replicate; colors and shapes indicate experimental groups. (**C**) functional enrichment analysis of differentially regulated proteins. Bubble plots show Gene Ontology (GO) Biological Process enrichment for significantly up- and down-regulated proteins at AmB 0.125 µg mL⁻¹ and Venetin-1 25 µg mL⁻¹. Bubble size represents gene count, and colour indicates false discovery rate (FDR). Amphotericin B was associated with enrichment of metabolic processes among up-regulated proteins and translational and biosynthetic pathways among down-regulated proteins, whereas Venetin-1 predominantly induced mitochondrial, oxidative and detoxification-related processes (45, 46).

Principal component analysis (PCA) revealed distinct patterns of global proteome organization for the two compounds (Fig. 10A, B). In the case of amphotericin B, samples treated with 0.125 µg mL⁻¹ formed a clearly separated cluster, while samples exposed to higher concentrations (0.25 and 0.5 µg mL⁻¹) grouped closely with control samples, indicating limited global proteomic changes under these conditions. In contrast, Venetin-1 treatment resulted in a concentration-dependent separation of experimental groups. Samples exposed to increasing concentrations (25, 50 and 100 µg mL⁻¹) formed progressively distinct clusters, indicating gradual proteome remodeling. The highest concentration (100 µg mL⁻¹) showed the greatest separation from control samples, reflecting the strongest global proteomic shift.

Consistent with PCA clustering, statistically significant differential protein regulation for amphotericin B was detected exclusively at 0.125 µg mL⁻¹. Functional enrichment analysis of up-regulated proteins revealed overrepresentation of metabolic and energy-related processes, including purine ribonucleotide biosynthesis, tricarboxylic acid cycle, proton transport and aerobic respiration (Fig. 10C). Down-regulated proteins were predominantly associated with translation and biosynthetic processes, including macromolecule and nitrogen compound biosynthesis. For Venetin-1, functional enrichment analysis performed at 25 µg mL⁻¹ revealed enrichment of processes related to mitochondrial function, oxidative metabolism, detoxification and reactive oxygen species processing among up-regulated proteins. Only a limited number of biological processes were significantly enriched among down-regulated proteins.

Together, these results indicate that amphotericin B and Venetin-1 induce distinct proteomic response patterns. Amphotericin B is associated with a concentration-restricted response detectable at sublethal conditions, whereas Venetin-1 induces a concentration-dependent and progressively expanding proteomic remodeling.

Detailed datasets and full enrichment analysis outputs supporting these results are available in the Supplementary Information (Supplementary Tables S2–S5 and Supplementary Figs. S1–S12).

## Electrokinetic analysis of Venetin-1 and amphotericin B

**Fig. 11:**
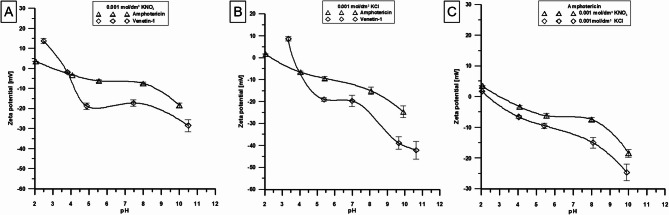
Dependence of ζ potential of: (**A**) Venetin-1 and AmB in the pH function in 0.001 mol/dm^3^ KNO_3_ solutions; (**B**) Venetin-1 and AmB in the pH function in 0.001 mol/dm^3^ KCl solutions; (**C**) AmB in the pH function in 0.001 mol/dm^3^ KCl and 0.001 mol/dm^3^ KNO_3_ solutions.

The pH dependence of the zeta potential for Venetin-1 and amphotericin B reveals pronounced differences in the electrokinetic behaviour of the two compounds (Figs. 11A, B). For both substances, the zeta potential shifts from positive values under acidic conditions to negative values with increasing pH. However, the magnitude of these changes and the sensitivity to the type of electrolyte anion differ significantly between the two systems. In the case of Venetin-1, a sharp decrease in the zeta potential is observed in the pH range of 3–5, followed by a pronounced plateau under near-neutral conditions and a further strong decrease in alkaline media. Venetin-1 reaches significantly more negative zeta potential values than AmB, particularly in the presence of chloride ions (Cl⁻). The observed zeta potential values indicate a strong dependence on both pH and electrolyte composition. AmB exhibits a milder and more gradual evolution of the zeta potential as a function of pH, with values remaining less negative over the entire investigated range. Under acidic conditions, the zeta potential assumes positive or near-zero values, while it gradually decreases and becomes negative with increasing pH (Fig. 11C). The zeta potential of AmB remains within a moderate range, from approximately + 15 mV to − 25 mV. A clear influence of electrolyte type on the electrokinetic characteristics of amphotericin-containing systems is observed (Fig. 11C). In the presence of chloride ions (Cl⁻), the zeta potential reaches more negative values under neutral and alkaline conditions than in solutions containing nitrate ions (NO₃⁻).

AmB exhibits positive electrophoretic mobility in an acidic environment (pH ≈ 2) in both KNO₃ and KCl solutions, indicating positive mobility values under strongly acidic conditions (Table 2). With increasing pH, a reversal of the mobility sign is observed, and the electrophoretic mobility becomes negative over neutral and alkaline pH ranges. The transition occurs gradually across the investigated pH range. Across the entire pH range, electrophoretic mobility values are consistently more negative in KCl solutions than in KNO₃ solutions. These differences become particularly pronounced under neutral and basic conditions. In KCl, AmB reaches higher absolute electrophoretic mobility values compared with KNO₃. Conductivity measurements reveal higher specific conductivity values for KCl solutions than for KNO₃ solutions, especially at extreme pH values. The differences in conductivity are observed consistently across the investigated pH range. A comparison of electrophoretic mobility and specific conductivity values for Venetin-1 and AmB shows that Venetin-1 exhibits higher absolute electrophoretic mobility values than AmB in the same electrolyte solutions.

**Table 2:** Electrophoretic mobility, conductivity, and pH of amphotericin B in 0.001 mol/dm^3^ KNO_3_ and 0.001 mol/dm^3^ KCl.

pH	Electrophoretic mobility [ms/Vs]	Conductivity [mS/cm]
0.001 mol/dm^3^ KNO_3_		
2.06	0.4527	6.04
4.08	-0.1868	0.394
5.56	-0.4485	0.140
8.01	-0.5429	0.201
10.01	-1.464	0.521
0.001 mol/dm^3^ KCl		
2.04	0.2612	4,92
4.04	-0.4227	0.124
5.43	-0.7602	0.138
8.08	-0.9701	0.152
9.91	-1.589	1.24

## Discussion

Fungal diseases result in millions of deaths each year, and the emergence of resistance to the few available antifungal agents exacerbates this problem. Invasive fungal infections are associated with immunosuppressive therapy, such as chemotherapy or solid organ transplantation, as well as hereditary or acquired immunodeficiencies. However, the most significant factors are considered to be the increasing number of patients with various immunocompromised conditions and the difficulties in diagnosing fungal infections^50–52^. Furthermore, the increasingly widespread use of invasive devices contributes to these infections^53^. Progress in medicine, including the use of immunosuppressants, glucocorticosteroids, antiretrovirals, and commonly used antibiotic therapies, significantly increases the incidence of candidiasis^34,54^. To date, approximately 150 species of *Candida* have been identified, of which about 20 are known to be pathogenic to humans^55,56^. However, *C. albicans* is responsible for approximately 70% of fungal infections worldwide, making it a major pathogen of humans^52,57^.

Amphotericin B exerts its fungicidal effect through three mechanisms: binding to ergosterol, disruption of homeostasis and oxidative stress induction^53,58,59^. Upon binding to ergosterol in the cell membrane, this antibiotic induces the formation of ion channels, resulting in the loss of protons and monovalent cations, which in turn leads to membrane depolarisation and, consequently, concentration-dependent cell death^60–62^.

In our studies, changes in the cell wall following incubation with AmB involved not only loss of cell wall integrity but also emergence of other altered forms, including cells with a thickened or irregular cell wall, signs of impaired cell division, chains of undivided cells, and the occurrence of giant cells. All of these morphological changes were also observed after Venetin-1 administration. The highest concentration of AmB resulted in more pronounced cell deformation compared to the changes induced by the highest concentration of Venetin-1. In both cases, the fungal cell wall was the target of the active substance.

The fungal cell wall is a two-layer structure. The outer layer is made up of mannoproteins (mannans and associated proteins), which constitute 38 to 40% of the dry weight of the cell wall. The inner (skeletal) layer is made of β-glucan, which is the main component of the fungal cell wall, constituting 50 to 60% of the dry weight of the cell wall, and chitin (2%)^63^. These polysaccharides are responsible not only for cell rigidity, shape, and protection against environmental stressors but also for the recognition of fungal cells by host immune cells. Chitin and β-glucan are two key fungal cell wall pathogen-associated molecular patterns (PAMPs) recognised by pattern recognition receptors (PRRs) expressed on the surfaces of innate immune cells, such as monocytes, macrophages, neutrophils, and dendritic cells^39,64^. In order to activate the immune system, β-glucan must be unmasked through the removal of mannoproteins. Compounds that result in exposure of β-glucan recognisable to the immune system may be valuable antifungal drugs or may be used synergistically with already available antibiotics^65^. In our research, we demonstrated that Venetin-1 causes the exposure of both the glucan and chitin layers in *C. albicans* cells, as evidenced by the over 6- and 3-fold increases in the average fluorescence of these components, respectively, compared to the control. Similar effects of β-glucan externalisation was observed by Suchodolski et al.^39^ after treatment of *C. albicans* with the lipopeptide surfactin. Comparable results were reported by Wheeler and Fink^66^ for *C. albicans* cells treated with sub-inhibitory concentrations of the antibiotic caspofungin. *C. albicans* SC5314 was also reported to exhibit increased β-glucan exposure in the presence of fluconazole and AmB^67^. Our previous studies^17^ confirmed that wild-type *C. albicans* strains also experience externalisation of β-glucans after incubation with fluconazole (5–20 µg mL^− 1^). Although fluorescence microscopy showed more intense fluorescence of β-glucans in *C. albicans* cells after AmB treatment compared to control cells, the flow cytometry analysis did not show differences. The difference in fluorescence is due to the fact that Congo red also binds to chitin, which is visible in the photo through the strongly luminous intercellular septa (Fig. 7)^68^.

The data obtained from the flow cytometric analysis of the cell wall are confirmed by the infrared spectroscopy results. The spectra of *C. albicans* cells after treatment with Venetin-1, and to a much lesser extent with AmB, clearly indicate a reduction in the intensity of signals originating from cell wall polysaccharides, particularly mannans, in the outer layer (Fig. 9). This indicates structural changes or/and the destruction of this layer of the wall, which resulted in the exposure of its deeper layers – β-glucan and chitin.

Qualitative and quantitative changes within the yeast cell wall translate into changes in the nanomechanical properties of the wall. The AFM analysis revealed a decrease in the Young’s modulus of cells treated with Venetin-1 and AmB, suggesting an increase in cell wall elasticity. Similar changes in the cytomechanical properties of yeast cells were observed by Ma et al.^69^ after exposure to a *S. sanguinis* bacteriocin. In turn, in their studies of *C. albicans* mutants, Hasim et al.^65^ observed a correlation between the phenomenon of β-glucan layer exposure and an increase in cell wall surface roughness and a decrease in its elasticity. In our studies, an inverse relationship regarding elasticity was observed, but we confirmed that the treatment with the Venetin-1 componud (previously called protein–polysaccharide fraction) increased the average roughness of the *C. albicans* cell wall surface compared to the control cells (R_a_ of control cells − 2.48 nm, R_a_ of tested cells − 9.94 nm)^12^. According to Ma et al.^69^, changes in cell surface properties, such as increased elasticity, increase the possibility of cell deformation and sensitivity to damage leading to cell death.

The SEM analysis facilitated comparison of the cell morphology of the analysed yeasts. The effects were similar after incubation with both Venetin-1 and AmB (Fig. 4). Many cells had numerous division scars located at one pole of the cell. Division disorders were also observed, where two mature cells could not separate from each other and remained attached. AmB disrupts the formation of a functional cell wall in young cells, thereby directly inhibiting the proliferation process. Similar effects after AmB treatment have also been observed by other researchers using SEM imaging^70^. This drug is also known to drastically inhibit the formation of germ tubes, a process necessary for the transformation of *C. albicans* into the hyphal form. An additional mechanism of action of the drug is the “sterol sponge” effect. AmB has the ability to form aggregates outside the membrane, which “pulls” ergosterol from the membrane, thus destabilising its structure. It can therefore be concluded that extramembrane aggregates kill yeast by extracting ergosterol from lipid bilayers^71,72^.

In *Candida* yeasts, apoptosis is a form of programmed cell death that can be triggered by various factors. These include stress, antifungal drugs such as AmB, or interaction with microporous surfaces, which leads to cell wall and membrane damage^73^ and, ultimately, fungal cell death. Apoptosis is associated with the accumulation of reactive oxygen species (ROS), depolarisation of the mitochondrial membrane, and activation of metacaspases^74^. For example, results indicate that aureobasidin A (AbA) potently induces apoptosis in *C. albicans* via mitochondrial dysfunction, making it a promising candidate in the fight against azole-resistant strains^75^.

AmB causes a rapid increase in reactive oxygen species (ROS), which, when present, lead to DNA fragmentation and disrupt the normal cell cycle^76^. It is known that AmB causes oxidative damage to cells through the formation of free radicals, which consequently leads to increased cell membrane permeability. In the case of Venetin-1, signs of oxidative stress have also been confirmed and described, contributing to the same effect^77^.

Fungal cell death following AmB and Venetin-1 treatment resulted from both necrosis and apoptosis (Fig. 3). Necrotic cells stained pink, and apoptotic cells with characteristically fluorescent nuclear material were observed. Fragmented nuclear material fluorescing blue-white was clearly visible in both cases, and evidence of *C. albicans* cell apoptosis following Venetin-1 administration has been described in our earlier publication^36^. It is known that AmB has the ability to induce apoptosis in *C. albicans* biofilms and in *C. krusei* and *C. parapsilosis* biofilms. At the same time, histone deacetylase (HAD) inhibitors have been shown to potentiate drug action and sometimes eliminate subpopulations of spores, confirming that histone acetylation can activate apoptosis in these cells^77^. In addition, studies have shown that programmed cell death is cell-type specific in yeast, namely filamentous *C. albicans* cells are more resistant to amphotericin B-induced programmed cell death than their blastospore counterparts^78^. The decrease in the OD value was associated with a decrease in the viability of *C. albicans* cultures after the action of Venetin-1 and AmB.

*Candida* exhibits cellular changes reminiscent of mammalian apoptosis. Many analyses focus on this similarity, but there are also certain differences. In the case of pathogenic organisms, these differences are encouraging, as they open up new research opportunities that can be used in the design of new drugs^79^. In fungi, apoptosis often leads to necrosis. High doses of a chemical agent can induce necrosis rather than apoptosis. The *Candida* cell wall, when degraded, releases toxins, which pose a significant threat to the host^80^. Therefore, the goal of new antifungal drugs is to induce apoptosis in *Candida* to destroy the fungi without releasing toxins.

Comparative analysis of proteomic responses revealed clear differences in the global cellular effects induced by amphotericin B and Venetin-1 (Table 3). The present SWATH-MS data demonstrate that amphotericin B induced detectable proteomic changes only at the lowest tested concentration, consistent with a concentration-restricted response under the applied experimental conditions. In contrast, Venetin-1 triggered a concentration-dependent remodeling of the *C. albicans* proteome. Increasing concentrations were associated with a progressive expansion of affected protein groups and functional categories, indicating gradual perturbation of cellular homeostasis rather than a single dominant response pathway.

**Table 3:** Comparative proteomic response of *Candida albicans* to amphotericin B and Venetin-1. The table summarises key differences in the extent and characteristics of proteomic changes induced by both compounds under the tested conditions. Venetin-1 exhibited a concentration-dependent response across multiple protein groups, whereas amphotericin B induced detectable changes only at the lowest concentration.

Feature	Amphotericin B (AmB)	Venetin-1
Proteomic response detected	Observed at 0.125 µg mL⁻¹	Observed at 25, 50 and 100 µg mL⁻¹
Dose-dependence	Not detected under tested conditions	Concentration-dependent pattern observed
Number of differentially regulated proteins	Lower number of proteins	Increasing number with concentration
Functional enrichment	Detected at 0.125 µg mL⁻¹	Detected across concentrations
Ribosome-related proteins	Predominantly down-regulated	Differentially regulated
Metabolic pathways	Limited representation	Broad representation
Mitochondrial processes	Moderately represented	Enriched among up-regulated proteins
Stress-related processes	Detected	Increasing representation with concentration
Proteome remodelling pattern	Restricted to specific conditions	Progressive across concentrations
Shared vs. specific proteins	Not identified	Core and concentration-specific proteins

Functional enrichment analysis further supports this distinction. Amphotericin B was primarily associated with downregulation of translational and biosynthetic processes accompanied by activation of selected metabolic pathways, consistent with a stress-adaptive response. In contrast, Venetin-1 treatment was linked to processes related to mitochondrial activity, oxidative metabolism and detoxification, suggesting involvement of multiple interconnected cellular pathways. Importantly, the limited number of significantly enriched processes among down-regulated proteins in Venetin-1-treated cells indicates a more selective regulatory effect, rather than global suppression of cellular functions.

Taken together, these findings indicate that proteome-scale analysis captures distinct modes of cellular response rather than differences in antifungal activity alone. While amphotericin B induces a restricted and condition-dependent proteomic shift, Venetin-1 is associated with a broader, concentration-dependent reorganization of cellular processes. This supports further investigation of Venetin-1 as a compound affecting multiple aspects of fungal cell physiology.

The electrokinetic behaviour of particles in a suspension is a key factor governing colloidal stability and the rheological properties of dispersed systems^81^. In formulations containing AmB, a compound characterised by strong amphiphilicity and limited aqueous solubility, electrokinetic stability is of particular importance due to the pronounced tendency of AmB molecules to undergo self-aggregation. The aggregation state of AmB is known to strongly influence its toxicological profile: monomeric forms exhibit higher selectivity toward ergosterol in fungal cell membranes, whereas supramolecular aggregates show increased affinity for cholesterol-containing host cell membranes, leading to enhanced cytotoxicity^82^. Aggregation processes are accompanied by changes in particle surface charge and potential distribution, which are directly reflected in zeta potential values and may promote system destabilisation. Consequently, zeta potential constitutes a key parameter for assessing the physicochemical stability of AmB-containing formulations, enabling quantitative characterisation of electrostatic interactions responsible for both particle aggregation and aggregation-mediated modulation of toxicity. The markedly different electrokinetic behaviour observed for Venetin-1 and AmB can be attributed to differences in their chemical structures and modes of interaction with electrolyte ions. The more negative zeta potential values attained by Venetin-1, particularly in alkaline media, indicate stronger electrostatic stabilisation and suggest a relatively high electrokinetic stability of Venetin-1 based systems. This behaviour is especially pronounced in the presence of chloride ions, pointing to efficient anion adsorption at the particle solution interface. In contrast, the limited decrease in zeta potential observed for AmB across the investigated pH range is consistent with its well-documented tendency to form supramolecular aggregates. The weaker sensitivity of amphotericin’s zeta potential to the nature of the anion suggests that aggregation in this system is governed predominantly by hydrophobic interactions and molecular self-organisation rather than classical electrostatic stabilisation mechanisms. The stronger response of Venetin-1 to the presence of Cl⁻ and NO₃⁻ ions, particularly under alkaline conditions, indicates a significant contribution of specific ion effects, potentially consistent with the Hofmeister series. For amphotericin, the comparatively smaller differences between KCl and KNO₃ solutions confirm the dominant role of aggregation phenomena over electrolyte-specific electrokinetic effects. From a pharmaceutical formulation perspective, these findings are highly relevant. Venetin-1 forms electrokinetically more stable systems, especially under alkaline conditions, whereas the control of pH and electrolyte composition is crucial for AmB primarily for limiting aggregation and the associated toxicity rather than for achieving high colloidal stability.

Fungi, like humans, are eukaryotic organisms, and most of their cellular processes are identical to those in humans. This makes finding a molecular target that will destroy the fungus without damaging patient’s cells extremely difficult. Due to this similarity, scientists have very few unique structures they could target. These are primarily ergosterol in the cell membrane and glucan in the cell wall.

In summary, it can be stated that the new Venetin-1 nanoparticle obtained from the coelomic fluid of *D. veneta* causes the reorganization of the cell wall structures of the

*C. albicans* fungus to a greater extent than amphotericin B, which antifungal mechanism is mainly related to the interaction with the cell membrane. Both compounds significantly reduced the survival of *C. albicans *cells in culture and induced cell death through apoptosis and necrosis. In addition, Venetin-1 creates stable electrokinetic systems important for formulation. However, differences were observed in the proteome of yeast cells treated with both compounds. Venetin-1 appears to be a promising compound for further analysis of its mechanism of action as a potential antifungal drug showing no cytotoxicity on normal human cells.

## Supplementary Information

Below is the link to the electronic supplementary material.

Supplementary Material 1

Supplementary Material 2

Supplementary Material 3

Supplementary Material 4

Supplementary Material 5

Supplementary Material 6

Supplementary Material 7

Supplementary Material 8

## Data Availability

Proteomics data have been deposited in the PRIDE repository under the number PXD074441. Original microscopic images, FTIR spectra and other data are available from the corresponding author on reasonable request.
