# Supplementary material for: Anti-Candida albicans effect of the new antifungal compound Venetin-1 obtained from earthworms versus amphotericin B
Source: Sci Rep. 2026 May 30;16:24761. doi: 10.1038/s41598-026-52961-2 (PMC13457962; doi:10.1038/s41598-026-52961-2)
Supplement: Supplementary file 1 [file 41598_2026_52961_MOESM1_ESM.pdf]

Supplementary Figures

Supplementary Figures – Downregulated proteins

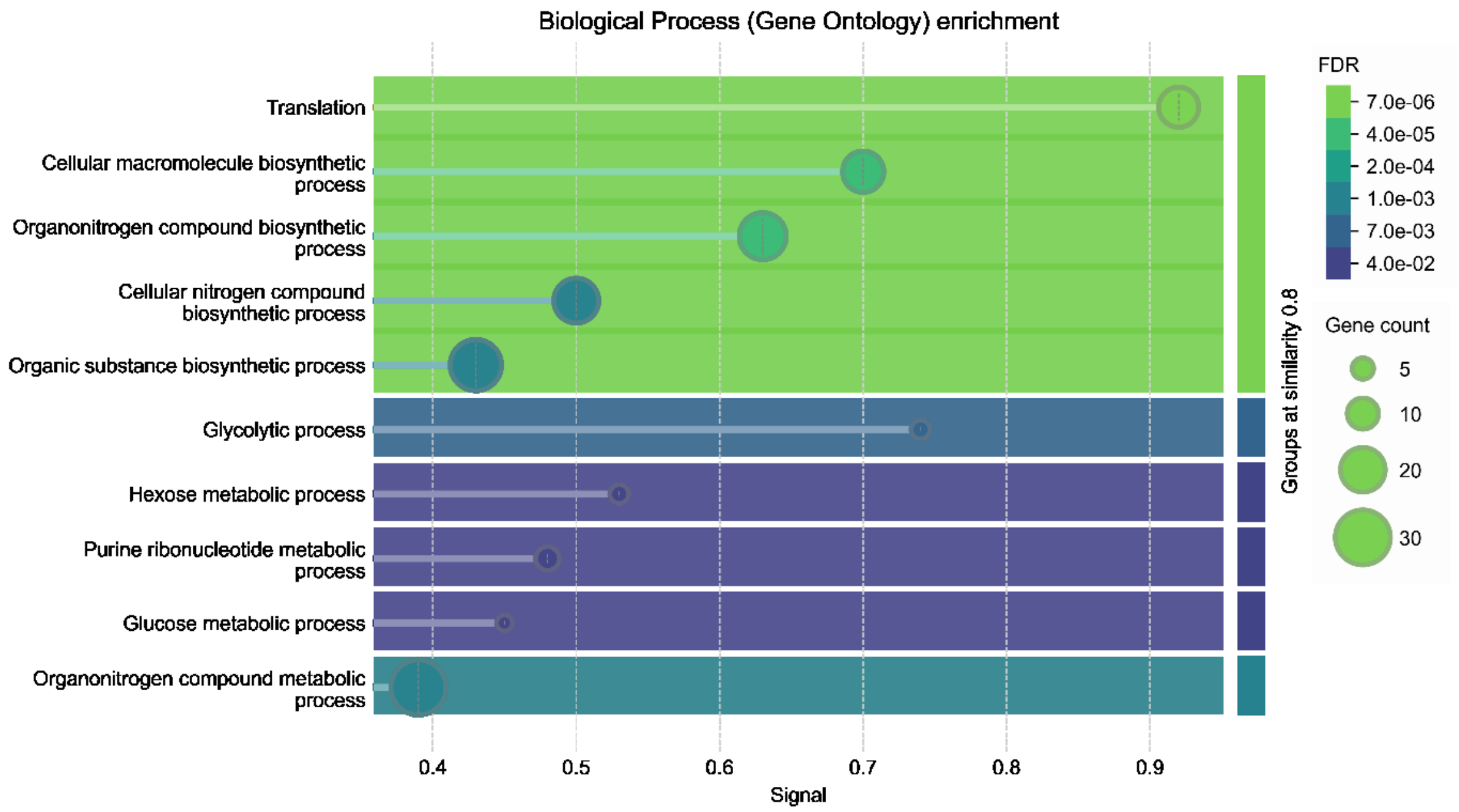

Figure S1. GO Biological Process enrichment of downregulated proteins. Bubble plot representation of enriched biological processes derived from STRING analysis. Bubble size indicates protein count, while color intensity corresponds to FDR-adjusted significance values.

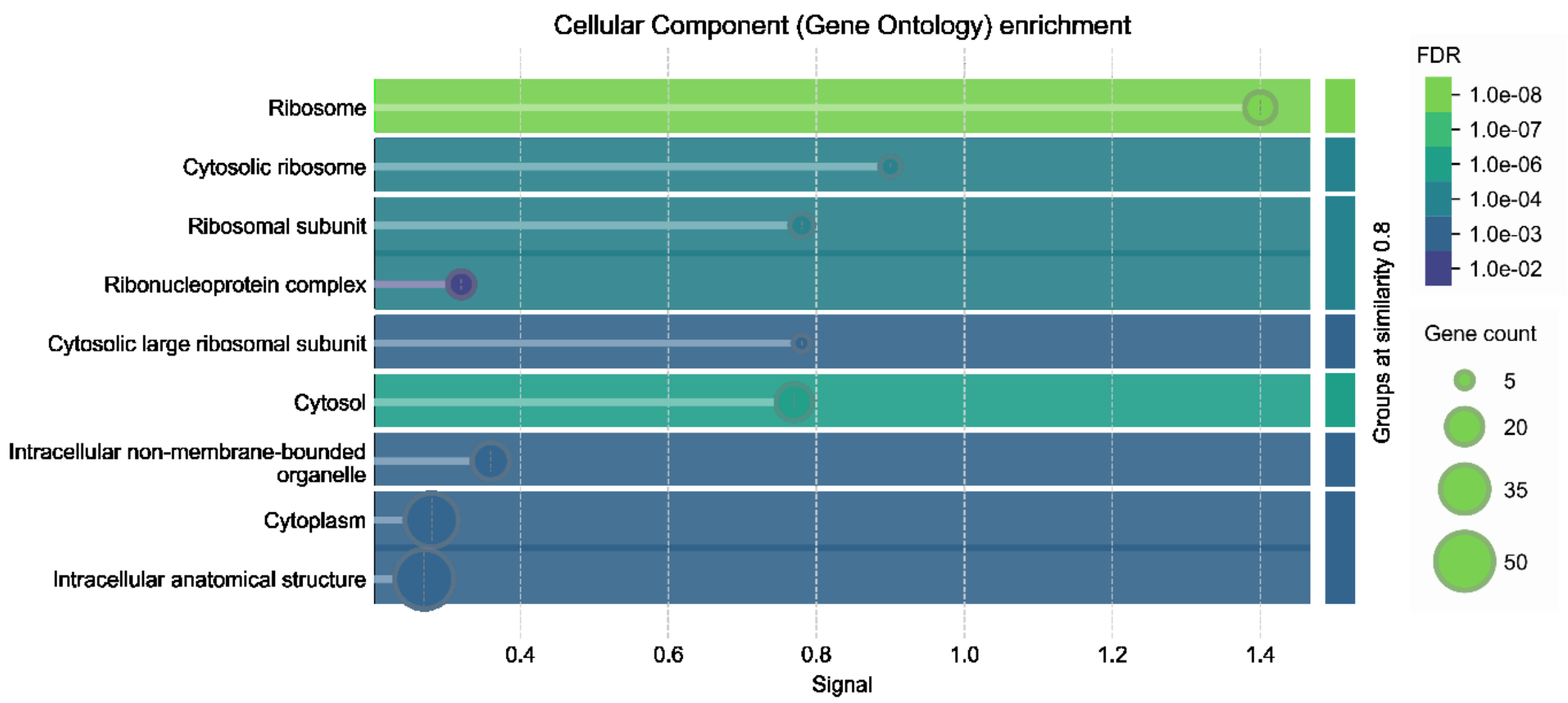

Figure S2. GO Cellular Component enrichment of downregulated proteins. Visualization of significantly enriched cellular localization terms associated with proteins decreased upon amphotericin B treatment.

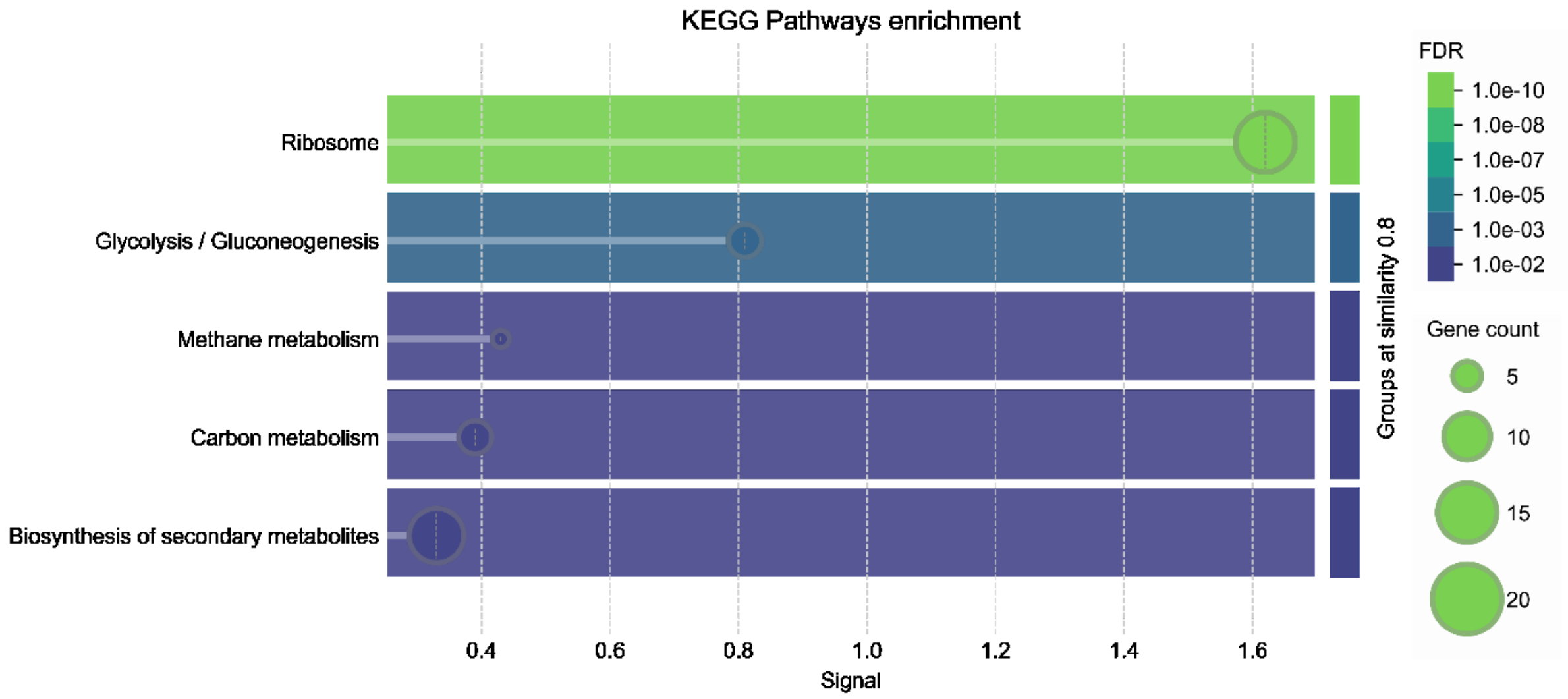

Figure S3. KEGG pathway enrichment of downregulated proteins. Dot plot summarizing enriched KEGG pathways. Bubble size reflects the number of mapped proteins and color scale represents FDR values.

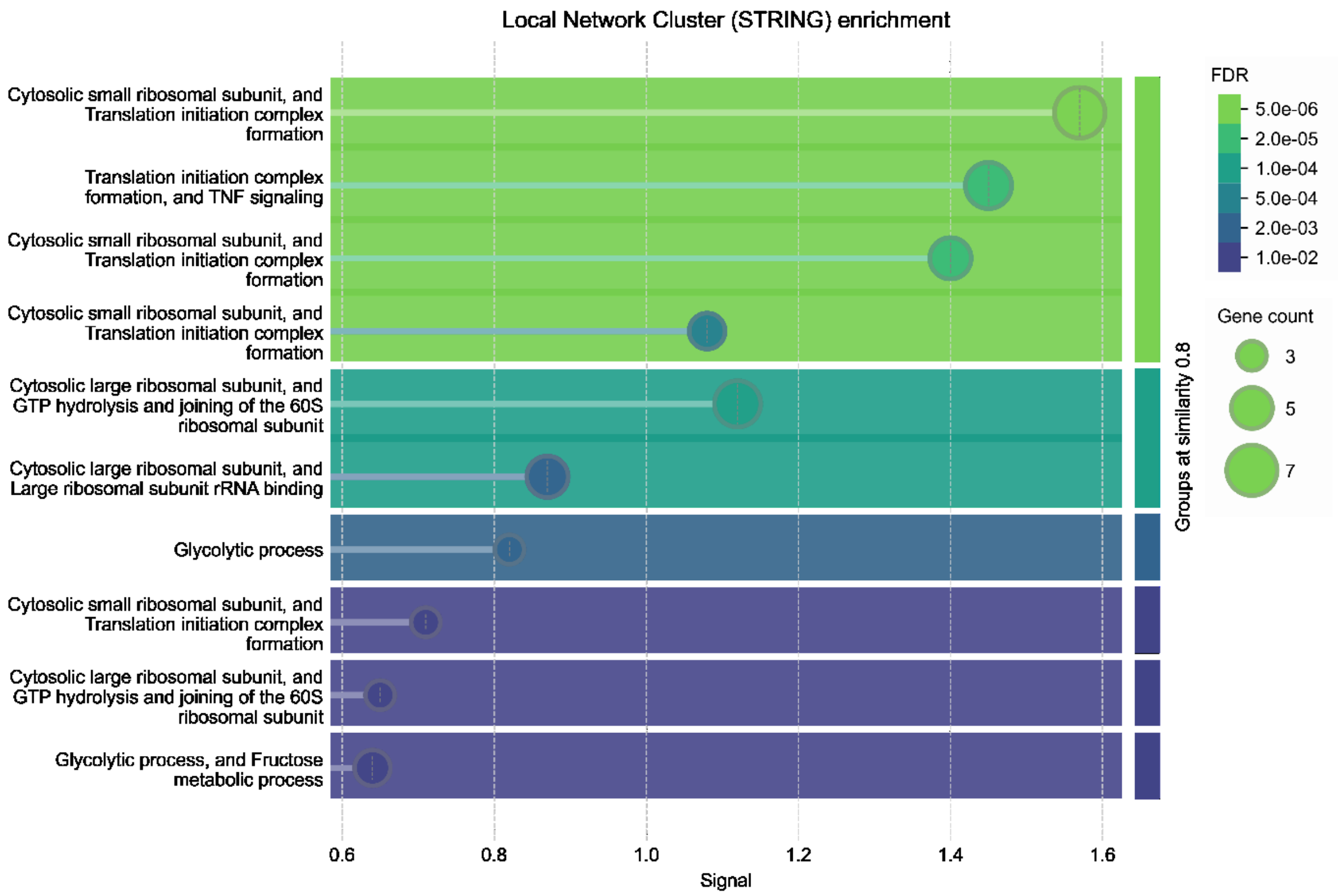

Figure S4. STRING local network clusters of downregulated proteins. Functional clusters identified from protein–protein interaction networks highlighting coordinated biological modules affected by treatment.

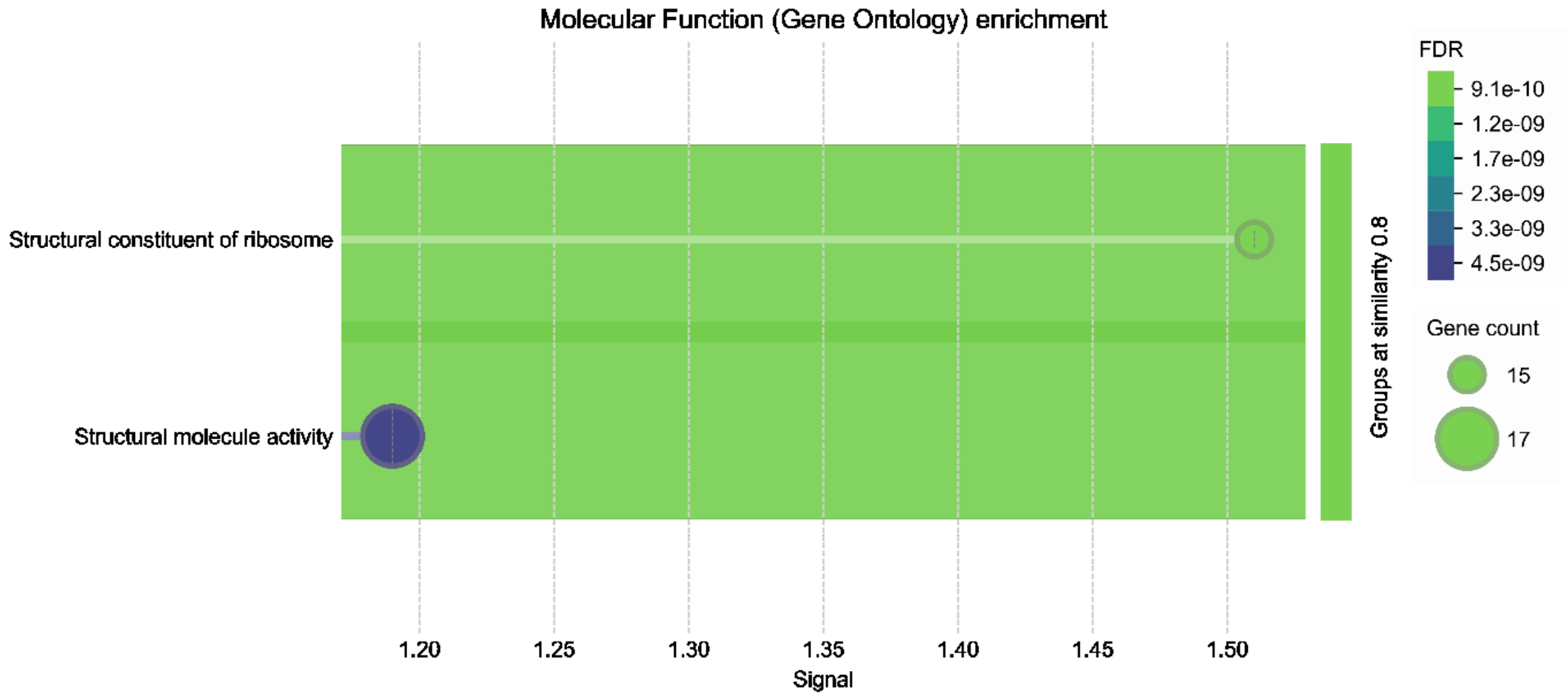

Figure S5. GO Molecular Function enrichment of downregulated proteins. Enrichment analysis describing biochemical activities and molecular functions associated with decreased protein abundance.

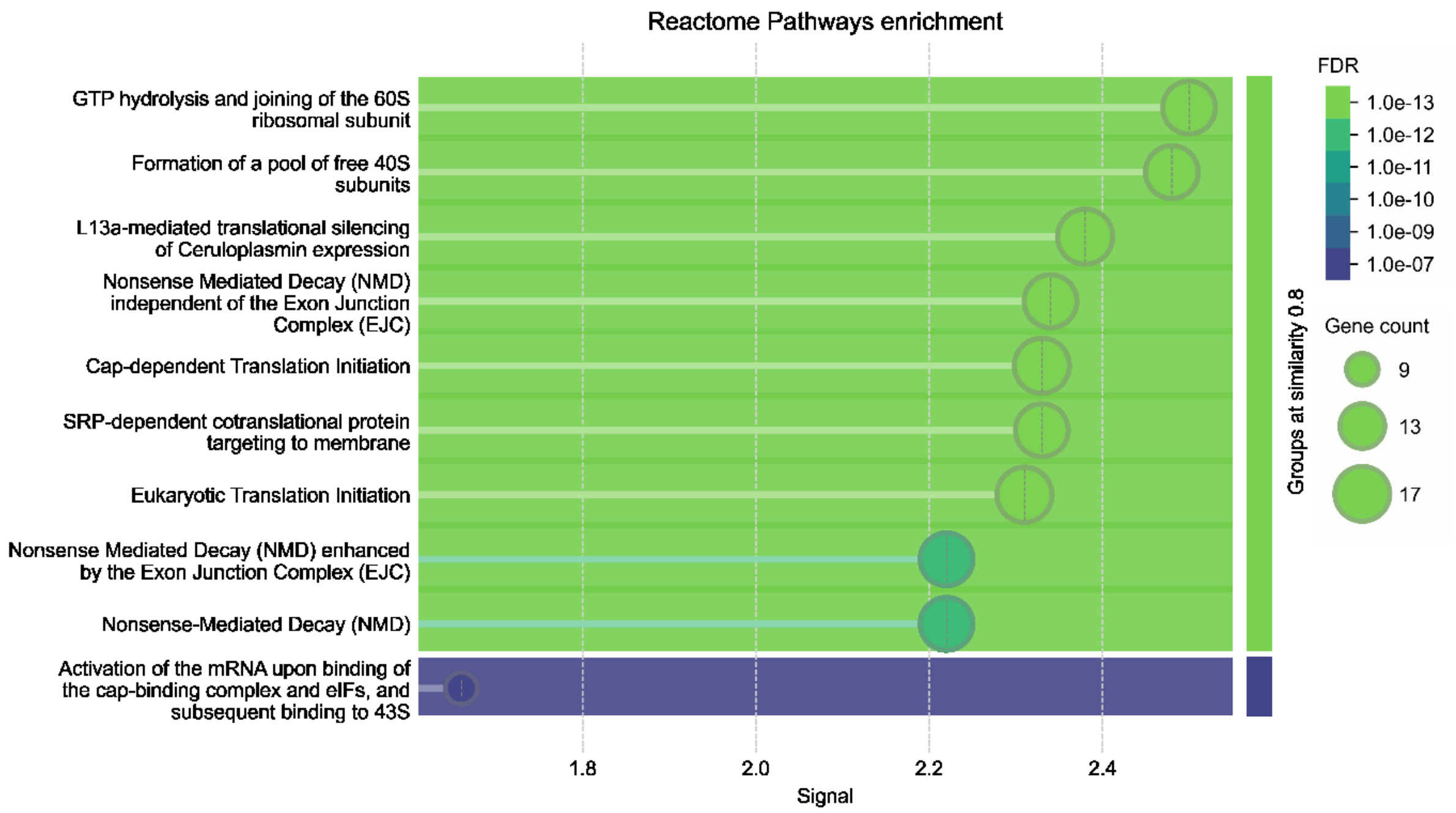

Figure S6. Reactome pathway enrichment of downregulated proteins. Overview of enriched Reactome pathways identified among downregulated proteins.

Supplementary Figures – Upregulated proteins

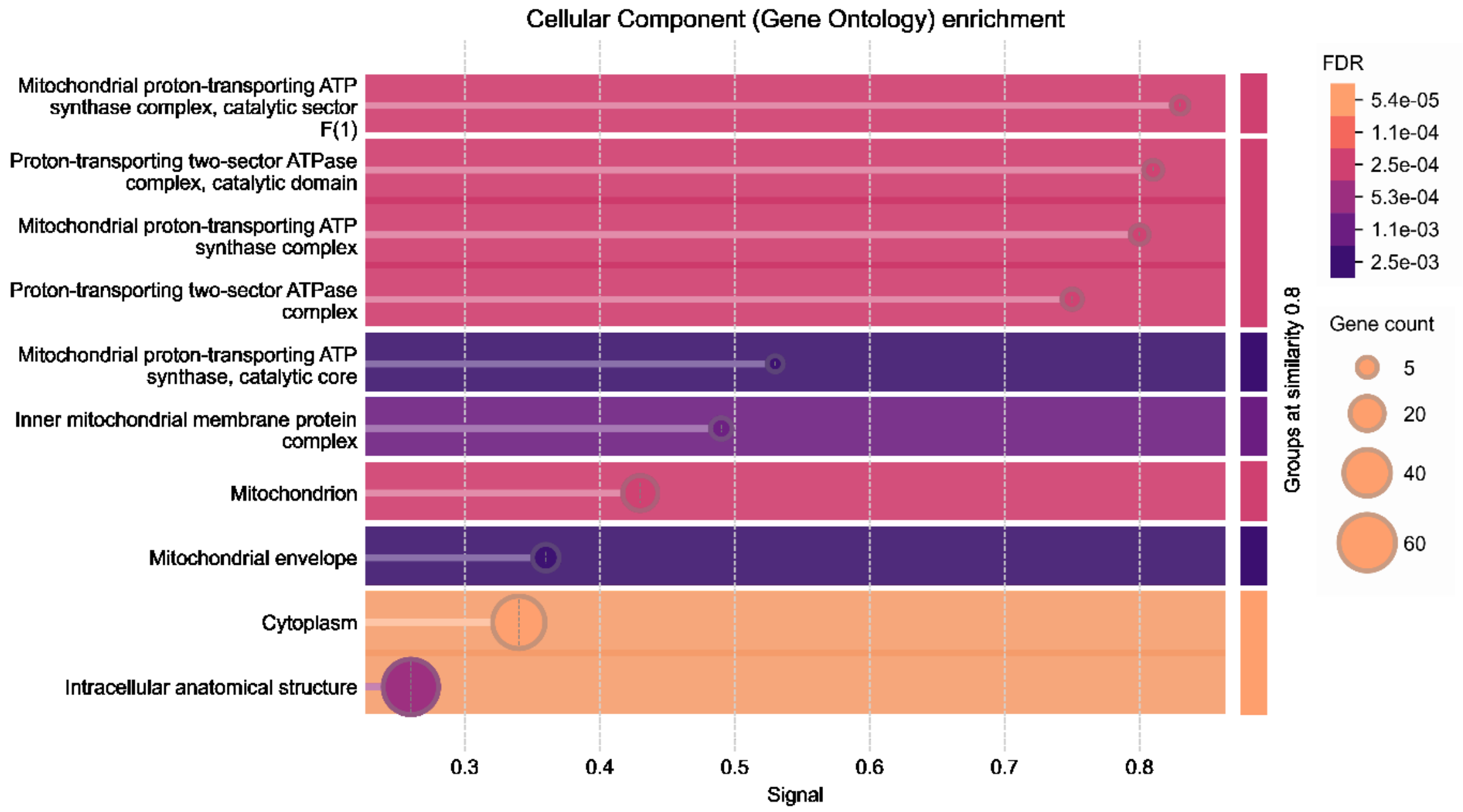

Figure S7. GO Cellular Component enrichment of upregulated proteins. Dot plot showing enriched cellular compartments associated with proteins increased following amphotericin B exposure.

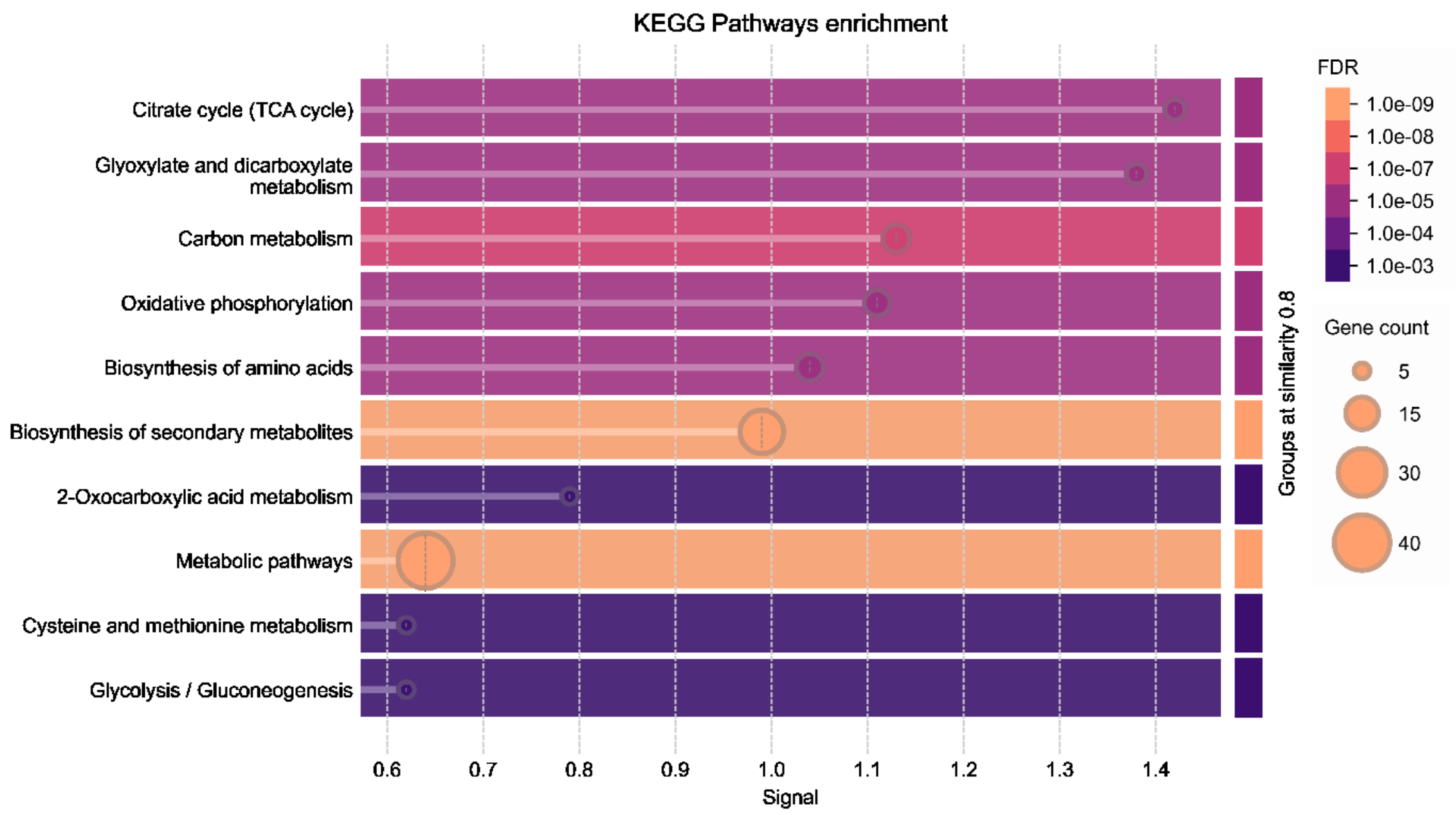

Figure S8. KEGG pathway enrichment of upregulated proteins. Visualization of KEGG pathways enriched among upregulated proteins. Bubble size corresponds to protein count and color indicates FDR significance

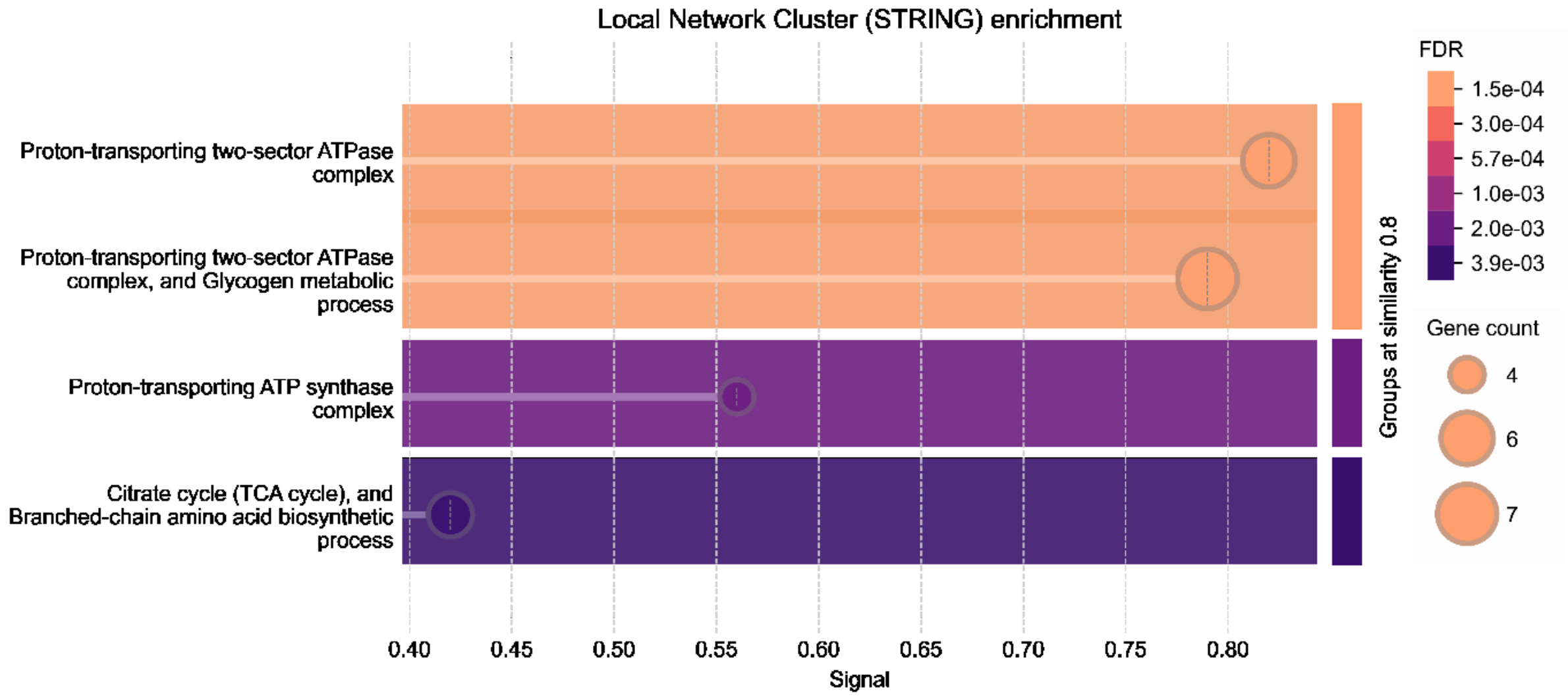

Figure S9. STRING local network clusters of upregulated proteins. Network-based clustering analysis highlighting functional modules enriched among proteins showing increased abundance.

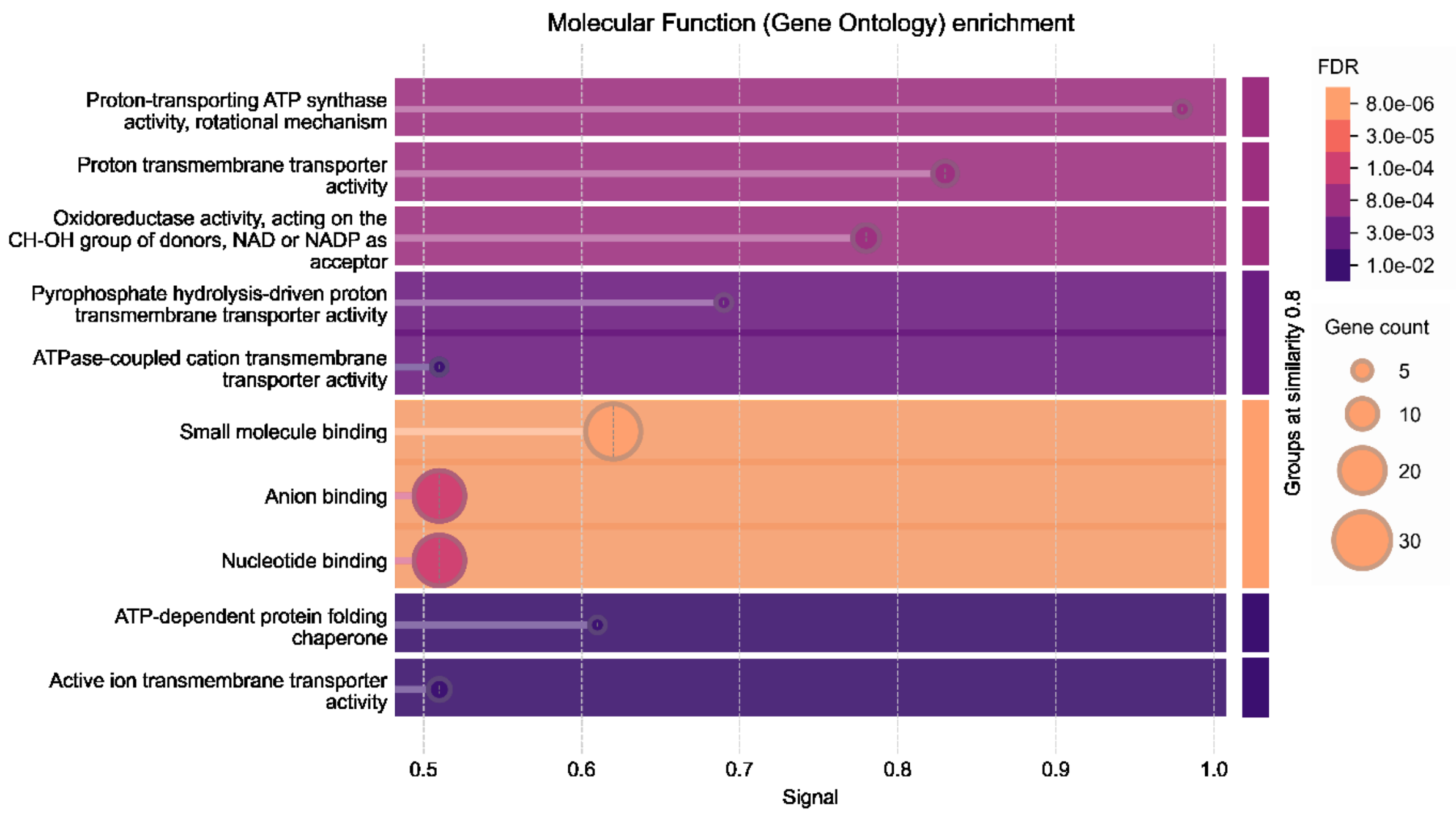

Figure S10. GO Molecular Function enrichment of upregulated proteins. Summary of molecular activities significantly enriched among upregulated proteins.

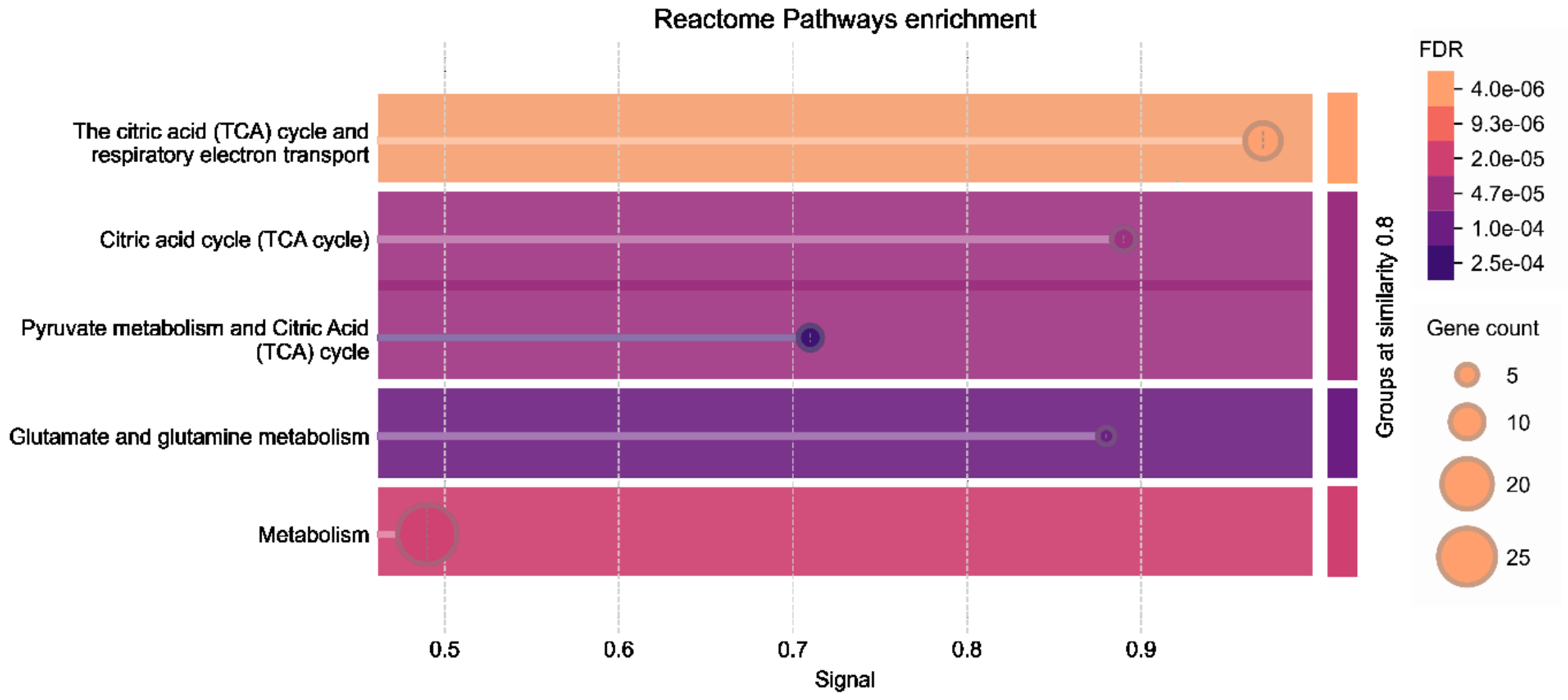

Figure S11. Reactome pathway enrichment of upregulated proteins. Reactome-based functional overview illustrating pathways associated with increased protein levels.

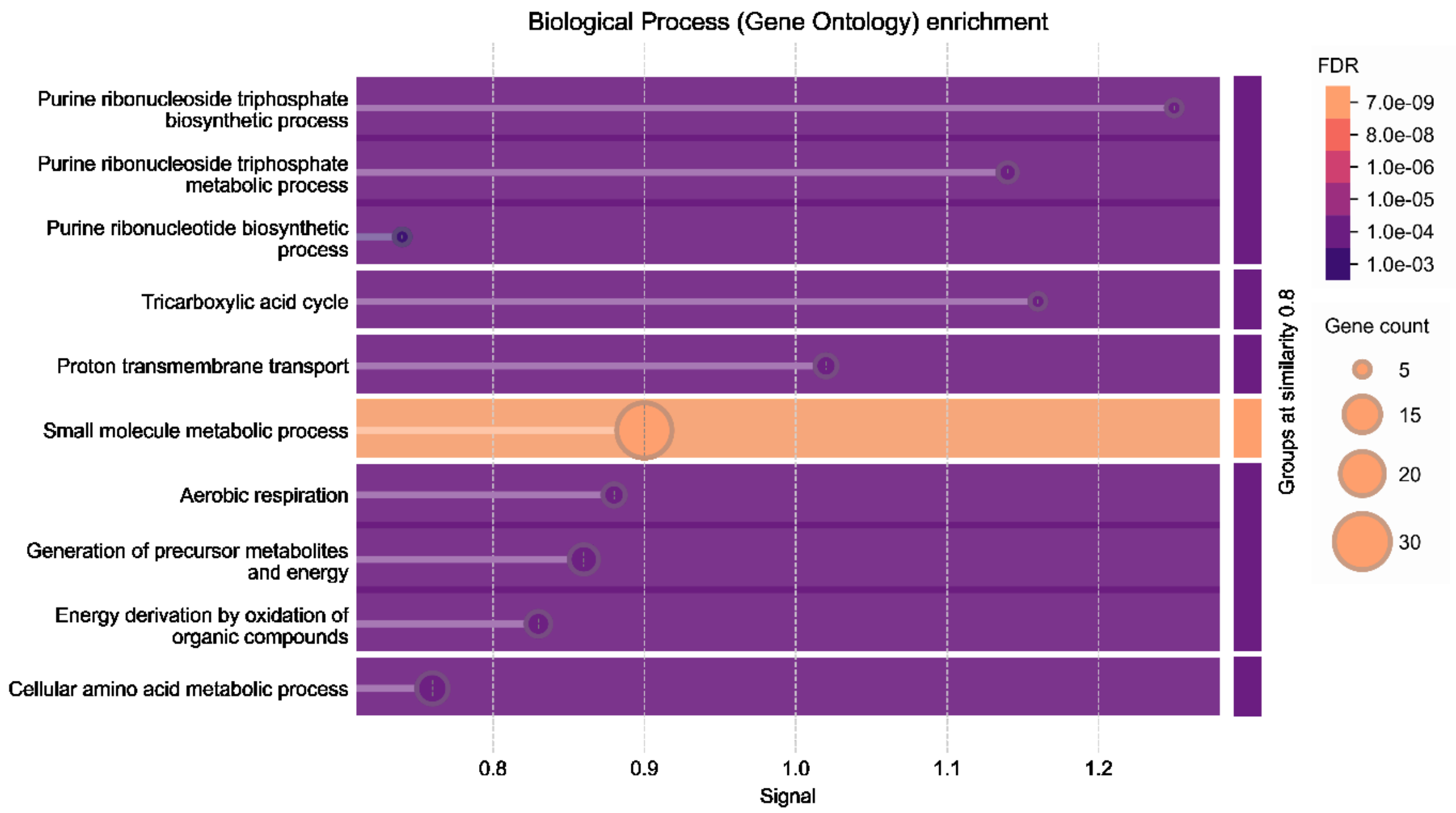

Figure S12. GO Biological Process enrichment of upregulated proteins. Bubble plot representation of enriched biological processes observed in response to amphotericin B treatment.
