# Supplementary material for: Anti-Candida albicans effect of the new antifungal compound Venetin-1 obtained from earthworms versus amphotericin B
Source: Sci Rep. 2026 May 30;16:24761. doi: 10.1038/s41598-026-52961-2 (PMC13457962; doi:10.1038/s41598-026-52961-2)
Supplement: Supplementary file 2 [file 41598_2026_52961_MOESM2_ESM.pdf]

Supplemetary Table S1. List of reagents

**Reagent name**	**Reagent abbreviation**
Sodium chloride	NaCl
Sabouraud medium	
Yeast extract	
Glucose	
Peptone	
Streptomycin sulphate	
Amphotericin B	AmB
Dimethyl sulfoxide	DMSO
Viacount reagent	
Glutaraldehyde	
Phosphate buffer	PBS
Saccharose	
Osmium tetroxide	OsO_4
Acetone	
Propidium iodide	PI
Hoechst 33342	
Calcofluor white	
Congo red	
Formaldehyde	
Wheat germ agglutinin conjugated with fluorescein isothiocyanate	WGA-FITC
Fc–hdectin-1	
Anti-human IgG Fc antibodies	
Sodium dodecyl sulfate	SDS
Tris(hydroxymethyl)aminomethane	TRIS
Dithiothreitol	DTT
Trypsin	
Formic acid	
Acetonitrile	
Potassium nitrate	KNO_3
Potassium chloride	KCl
Nitric acid	HNO_3
Potassium hydroxide	KOH
