# Supplementary material for: Anti-Candida albicans effect of the new antifungal compound Venetin-1 obtained from earthworms versus amphotericin B
Source: Sci Rep. 2026 May 30;16:24761. doi: 10.1038/s41598-026-52961-2 (PMC13457962; doi:10.1038/s41598-026-52961-2)
Supplement: Supplementary file 3 [file 41598_2026_52961_MOESM7_ESM.pdf]

Supplementary Tables legends

Supplementary Table S2. SWATH-MS quantitative proteomic dataset. Complete output of the SWATH-based proteomic analysis for *C. albicans* after amphotericin B treatment including protein accession numbers, quantitative fold changes, statistical significance values, and regulation status. This dataset was used as input for downstream functional and pathway enrichment analyses.

Supplementary Table S3. Functional enrichment analysis of downregulated proteins. STRING-based enrichment analysis performed on proteins significantly downregulated following amphotericin B treatment of *Candida* cells. The table includes enriched Gene Ontology (Biological Process, Cellular Component, Molecular Function), KEGG pathways, Reactome pathways, and STRING local network clusters. Reported parameters include enrichment statistics, false discovery rate (FDR), and associated protein identifiers.

Supplementary Table S4. Functional enrichment analysis of upregulated proteins. STRING enrichment results for proteins showing increased abundance after amphotericin B exposure. Enrichment terms span GO annotations, KEGG and Reactome pathways, and local STRING interaction clusters. Each entry contains enrichment scores, FDR-adjusted values, and protein lists assigned to the respective category.

Supplementary Table S5. SWATH-MS quantitative proteomic dataset
Complete output of the SWATH-based proteomic analysis for *C. albicans* after Venetin-1 treatment including protein accession numbers, quantitative fold changes, statistical significance values, and regulation status. This dataset was used as input for downstream functional and pathway enrichment analyses.
