# Supplementary material for: Anti-Candida albicans effect of the new antifungal compound Venetin-1 obtained from earthworms versus amphotericin B
Source: Sci Rep. 2026 May 30;16:24761. doi: 10.1038/s41598-026-52961-2 (PMC13457962; doi:10.1038/s41598-026-52961-2)
Supplement: Supplementary file 4 [file 41598_2026_52961_MOESM8_ESM.docx]

**Supplementary materials**

The list of reagents with full names and abbreviations is presented in Supplementary Table S1. The complete SWATH-MS quantitative proteomic dataset for *C. albicans* after amphotericin B treatment is provided in Supplementary Table S2. Functional enrichment analyses of downregulated and upregulated proteins, including Gene Ontology categories, KEGG and Reactome pathways, and STRING local network clusters, are presented in Supplementary Tables S3, S4 and visualised in Supplementary Figures S1–S12.  The complete SWATH-MS quantitative proteomic dataset for *C. albicans* after Venetin-1 treatment is provided in Supplementary Table S5.
